# How effective are video animations as information tools for patients and the general public? An updated systematic review

**DOI:** 10.3389/fdgth.2025.1717044

**Published:** 2026-01-02

**Authors:** Thirimon Moe-Byrne, Peter Knapp, Amber Lidster, Mim Ahamed, Hugh O'Hare, Su Golder, Jennie Lister, Joy Adamson

**Affiliations:** 1 Department of Health Sciences, University of York, York, United Kingdom; 2 Hull York Medical School, University of York, York, United Kingdom; 3 Sheffield Teaching Hospitals NHS Foundation Trust, Sheffield, United Kingdom

**Keywords:** video animations, information tools, patients, knowledge, attitudes and cognition, behaviours, albatross plot

## Abstract

**Background:**

Online and digital communications have changed information access, with many people using the internet for health information. Our 2022 systematic review showed that video animations can improve short-term patient and public knowledge but questions remained about their longer-term effectiveness, particularly for non-native speakers and those with low health literacy, and about their effects on attitudes, cognitions (e.g., self-perceptions) and behaviour.

**Methods:**

This review updates a previous systematic review on the effectiveness of video animations compared to other information formats. It includes randomised or quasi-randomised controlled trials, focusing on patients’ or public understanding of health topics. The same eligibility criteria and search strategy were used, without language restrictions, and multiple databases were reviewed to April 2025 (our 2022 review had searched from database inception to June 2021). Inclusion assessment, data extraction, and quality appraisal were conducted independently by two researchers. Findings are presented through narrative synthesis and albatross plots.

**Results:**

We included 87 publications (88 trials), including 50 trials new to this update, focusing on medical procedures (*n* = 40), condition management (*n* = 24) and public health (*n* = 24). The median trial sample size was 120 and trials had been undertaken in 28 different countries. Animations showed positive effects for knowledge [48/60 trials (80%)], attitudes and cognitions [28/53 trials (53%)] and behaviours [20/32 trials (63%)]Null effects were found in 18% studies assessing knowledge, 47% studies of attitudes and cognitions, and 34% studies of behaviour, with one negative effect each in knowledge (2%) and behaviour (3%). Overall, risk of bias was “high” (*n* = 37), “some concerns” (*n* = 35), or “low” (*n* = 16), often due to concerns about randomisation, blinding, small samples, missing data or unpublished protocols.

**Discussion:**

Video animations improve patient knowledge and behaviour in the short-term, with some positive effects on attitudes and cognitions. However, higher quality and larger randomised controlled trials are needed to evaluate longer-term outcomes, especially for individuals with low health literacy. Practitioners should consider incorporating animations into public health, health education and healthcare delivery while being mindful of current research limitations.

**Systematic Review Registration:**

https://www.crd.york.ac.uk/PROSPERO/view/CRD42024559912, PROSPERO CRD42024559912.

## 1 Background

Online and digital communications have become commonplace in many countries, with many people using the internet to obtain health information. As a result, there are opportunities to disseminate health information to patients and the public in a range of ways, potentially offering advantages for service providers and recipients alike.

Historically, information has been conveyed to patients through clinical consultations, information leaflets (with or without images), or short television films for some public health issues. However, patients may not always fully grasp the information being conveyed to them, potentially because of cultural and educational differences between healthcare providers and patients (1). This issue is particularly important for individuals with limited health literacy. This group of people may struggle to comprehend certain health information, and studies indicate that information that is too detailed or complex may deter people from participating in health evaluations such as screening (2).

The utilisation of digital technologies has opened new ways to deliver information to patients and their families, offering potential benefits. The SAWBO organisation, for instance, has produced numerous brief, animated videos in various languages covering public health subjects (3). Evidence shows that incorporating graphics and animations in information can increase both comprehension and the recall of facts about healthcare interventions (4, 5).

In 2022 we published a systematic review of the effectiveness of video animations (6), which included 38 trials and showed consistent positive effects of animations on patient knowledge, when compared to another intervention, such as printed information or in-person consultation. The review also showed some evidence of positive effects on patient attitudes and cognitions (such as satisfaction with information, self-confidence or perceived quality of life) and patient behaviour or intended behaviour (such as medicine taking or effective inhaler use). The 2022 review included evidence published up to June 2021, and we were aware of significant amounts of recent research activity in this rapidly evolving field. Furthermore, our published review included relatively few trials that evaluated patient behaviour or longer-term knowledge retention, and many of the included trials were small and none had included a cost-effectiveness evaluation. Therefore, the aim of this work was to update the previous review, to assess the effectiveness of video animations as information tools, when compared to other forms of provision.

## 2 Methods

The updated review protocol was registered with PROSPERO (https://www.crd.york.ac.uk/PROSPERO/view/CRD42024559912) and has been reported in accordance with PRISMA guidelines (7).

### 2.1 Data sources and searches

The aim of the searches was to identify trials that evaluated the effectiveness of video animations as information tools for patients or the public. The search strategy used in our previous review was revised slightly (6), in accordance with changes to database terminology, and was run in Medline (Ovid) and then adapted for other databases (see Supplementary Materials: Search Strategies).

The following databases were searched on 7th June 2024 and updated again on 24th April 2025: Medline, Medline in-Process, EMBASE, CINAHL Plus, Cochrane Database of Systematic Reviews and PsychINFO. All search results were de-duplicated using EndNote. Additionally, we performed both forwards- and backwards-citation searches through Google Scholar and the reference lists of newly included articles, and also undertook forwards-citation searches of the 38 trials included in the previous review. No language restrictions were applied.

### 2.2 Eligibility criteria

We used the same eligibility criteria as previously.

Participants were either individuals receiving healthcare services or members of the public being educated on public health, health promotion, health screening or illness prevention topics. To be eligible, studies had to employ a randomised or quasi-randomised controlled design, specifically comparing the effectiveness of a video animation (categorised as cartoons, avatars, “white board animations”, or animated 2D or 3D diagrams) against an alternative method of information delivery, such as printed materials, audio recordings, videos of actual people or health facilities, procedural videos or spoken information (including that delivered as part of standard care). We included trials evaluating animations as an alternative to another format, and those in which the animation was provided in addition to another format. When an animation was provided as well as usual care, and compared to usual care alone, we classified that trial as testing an animation as an additional provision.

Video animations of any length were eligible, and we included animations with or without a voiceover. Animations were eligible if they were part of a comprehensive information package, if the specific impact of the animation could be identified. However, we excluded studies that lacked a control group, examined hypothetical scenarios, or compared the animation against no information provision. For inclusion, trials had to assess outcomes in at least one of three categories: (i) knowledge; (ii) attitudes and cognitions, such as satisfaction with information received or self-confidence; (iii) health behaviours or intended behaviours, such as appointment attendance or condition self-management. We did not extract data on health outcomes, such as pain, mood or blood pressure, given the expected wide range of outcomes, which would often be context-specific, and because our primary focus was on the educational and psychological effects of the interventions.

### 2.3 Study selection

**Figure 1:**
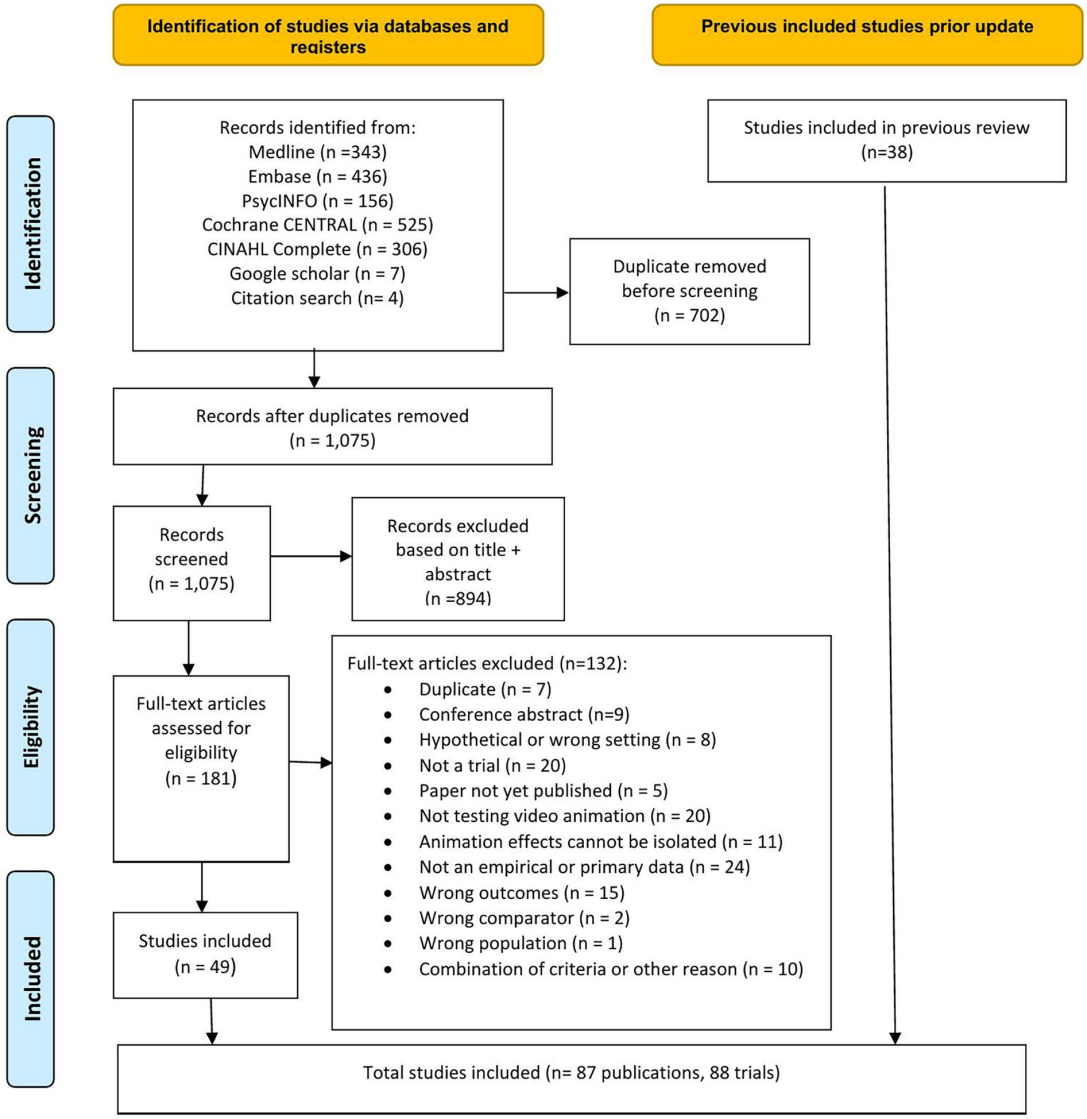
PRISMA flow chart.

The de-duplicated records were imported into Covidence software for screening (8). Two reviewers (two of MA, PK, AL) independently screened the titles and abstracts of all retrieved records against the predefined eligibility criteria. Two reviewers (two of TMB, PK, MA, AL, HO) then independently assessed the full texts of potentially relevant articles. Any disagreements were resolved through discussion or consultation with a third reviewer (see Figure 1 for the PRISMA flowchart). For studies where full text was unavailable through database searches, institutional access or open-access repositories, we contacted the corresponding author.

### 2.4 Data extraction

We used Covidence software to extract data, including basic study information, details of participants, the type of intervention and control arms(s), details of the intervention, and outcome data. One reviewer (MA, TMB, or PK) conducted the data extraction, which was then checked by a second reviewer. Any disagreements were resolved through consensus.

### 2.5 Quality assessment

We employed the Cochrane Risk of Bias Tool-2 (RoB-2) (9) to evaluate the methodological quality of each included trial using five key criteria: the randomisation process, deviations from intended interventions, missing outcome data, outcome measurement and the selection of reported results. For the included cluster trial, we included one extra domain “the identification or recruitment of participants into clusters”. One reviewer (TMB) conducted the risk of bias assessment, which was checked by a second reviewer (PK) and any discrepancies were resolved through consensus.

### 2.6 Data synthesis

The included trials were combined with the 38 trials from the 2022 publication.

Due to the degree of heterogeneity among included trials, particularly in terms of the intervention, comparators and patient populations, statistical meta-analysis was not feasible. Therefore, a narrative synthesis approach was used according to three pre-identified outcome categories (knowledge; attitudes and cognitions; behaviour).

We have taken reports of differences between trial arms of *p* < .05 as indicators of effect. When trials compared an animation to a control group and evaluated outcomes at multiple time points, resulting in inconsistent findings (for example, positive effects at one-time point and no difference at another), we have reported the overall findings as indicating some positive effects of animation. Conversely, if the results showed a combination favouring the control group at one time point and indicated no difference between animation and control at another, we reported this as some negative effects of the animation. Similarly, if the study reported individual results for various questionnaires related to one outcome category, for instance, if the outcome was statistically significant in 5 out of 10 knowledge measures, we indicated that there were some positive effects associated with the animation. Lastly, we counted the frequency of outcomes related to knowledge, attitudes and cognitions, and behaviours, categorising them as positive, somewhat positive, no difference, or negative.

To complement the narrative synthesis and provide a visual representation of the findings, we created six albatross plots (three reporting animations as an alternative format and three reporting animations as an additional format). Albatross plots require only a total sample size, corresponding *p*-value and direction of effect, and are a useful alternative to a traditional meta-analysis where availability of results is limited or there is variation in reporting between studies. The basic albatross plot is a scatter plot of 2-sided *p*-values (*X* axis) against study sample size (*Y* axis), with results separated along the *X*-axis by direction of effect. Contours on the plot visualise approximate effect sizes that would have resulted in the *p*-values shown. In this way, the plots allow a visual comparison of results in cases where meta-analysis is not feasible, though it should be noted that they are intended to serve as interpretive aids rather than precise effect estimators (10). Plots were generated using StataNow/MP 18.5 (11) using the *albatross* command, with the standardised mean difference (SMD) option chosen to generate the contours (12). Where trials reported results at multiple time points, plots included only the first post-intervention time point to ensure consistency. Similarly, when trials used different measurement tools to assess the same outcome, we selected the stated primary outcome or overall score, or chose the outcome most relevant to the outcome category (i.e., knowledge, attitudes and cognitions, behaviour). When a trial reported multiple measures (e.g., several knowledge items) and the primary outcome was not stated, we included results in the albatross plot if all relevant statistical results were the same (i.e., all statistically significant or all not significant) and, in the case of all significant results, used the most conservative of the reported *p* values. Also, when trials did not report the precise *p*-value and indicated it as *p* < 0.05 or *p* < 0.01, for example, we adopted a conservative estimate of 0.05 or 0.01 for analysis. Conversely, if the reported *p*-value exceeded 0.05 (for example, *p* > 0.05), we assigned a value of 1 to maintain consistency in our evaluation.

## 3 Results

### 3.1 Study characteristics

The study selection process is illustrated in the PRISMA flowchart (see Figure 1). We conducted a comprehensive database search using publication dates from July 2021 to April 2025 that identified 1,777 publications. After removing duplicates, we screened 1,075 unique titles and abstracts for relevance. Of these, 181 publications were deemed eligible for full-text review, of which 132 were excluded for specific reasons and 49 publications were included in the review. We have combined these findings with the 38 trials from our previous review (6). Consequently, our final analysis includes 87 publications, comprising 88 randomised controlled trials (RCTs), published between 1996 and 2025, including two RCTs from one publication (13).

The 87 publications (including 88 trials and 99 intervention arms) included 82 individual randomisation RCTs, two cluster RCTs, three quasi-RCTs, and one combination of RCT and quasi-RCT (see summary Tables 1–3 and full details Supplementary Tables 1–3). Study samples ranged from 30 to 16,716 participants and the 88 included trials recruited a total of 37,900 participants. The trials were conducted across a wide range of countries (https://datawrapper.dwcdn.net/fa0HR/9/), with 33 trials coming from either upper-middle-income countries (UMICs) or lower-middle-income countries (LMICs) (14), and the remainder coming from Organisation for Economic Co-operation and Development (OECD) countries. Overall, trials had been undertaken in 28 countries, most commonly the USA (15 trials). Australia and Turkey each contributed nine trials followed by Thailand with eight trials. The remainder included five trials each from Canada and the UK, and four from China, with the other 21 countries each contributing 1–3 trials (see Supplementary Tables 1–3). Studies were reported in English except for three trials which were reported in German (15), Korean (16), and Arabic (17), and which were translated for inclusion.

#### 3.1.1 Topic, style and length of the animations

3D animated diagrams were utilised in 12 trials (15, 18–28), while 2D animated diagrams were featured in 6 trials (29–34). One trial incorporated both 2D and 3D animated diagrams (35). Cartoon animations were used in 32 trials (5, 13, 36–64),while avatar apps were used in 2 trials (65, 66) and a whiteboard animation was used in four trials (67–70).

In five trials animation was used as part of a multimedia intervention (71–75) and 3D animation was included in four of those trials. The remaining 26 trials reported using video animation without specifying its type (4, 16, 17, 76–98). The duration of animations ranged from 27 s to 31 min (median 6 min), although the duration was not reported in 12 trials. The 87 publications covered a wide variety of topics and health settings, which are listed in Tables 1–3.

**Table 1:** Category 1 findings (explaining medical or surgical procedures).

Author, year	Country	study design	ROB	Sample size	Participants	Time points	Interventions vs. control	Knowledge	Attitude	Behaviour
Bowers et al. (71)	Canada	RCT	High	93	Adults undergoing first-time peripherally inserted central venous catheter (PICC), Hickman catheter, peripheral angioplasty with or without stenting, or endovascular aneurysm repair	During the consent process	Animation + Verbal vs. Verbal	↑	↑	—
Bozkul et al. (76)	Turkey	RCT (3 arms)	Some	60	Children undergoing planned surgery	Postoperative period (A)	Animation + Video vs. Short film + Usual care	—	®	—
							Animation + Video vs. Usual care	—	↑	—
Can et al. (93)	Turkey	RCT	High	156	Patients undergoing ureteroscopic lithotripsy	Post-intervention (A)	Video animated Information + Verbal and written information vs. Verbal and written information		↑	
Chanthawong et al. (64)	Thailand	RCT (3 arms)	Some	163	Adults due to have planned surgery	Immediately after visit (K), day of surgery (K); 1 day after visits (A)	In-person information + Animation vs. In-person information + Brochure	↔ ↔	↔	—
							In-person information + Animation vs. In-person information only	↑ ↑	↔	—
Cornoiu et al. (73)	Australia	RCT (3 arms)	High	61	Adults due to have planned knee arthroscopy	Day of surgery; 3-6 weeks post-surgery (K,A)	Animation vs. Verbal	↑ ↑	↔	—
							Animation vs. Pamphlet	↑ ↑	↑	
Degirmentepe et al. (26)	Turkey	RCT	High	70	Female patients with urinary incontinence	Post-intervention (A, B)	Video animation+ Written and verbal information vs. Written and verbal information only		↑	↑
Degirmentepe et al. (25)	Turkey	RCT	Some	80	Patients scheduled for Extracorporeal Shock Wave Lithotripsy (ESWL)	Post-intervention (A, B)	Video animation+ Written and verbal information vs. Written and verbal information only		↑	↑
Degirmentepe et al. (27)	Turkey	RCT	High	160	Patients undergoing Flexible cystoscopy	Post-intervention (A, B)	Video animation+ Written and verbal information vs. Written and verbal information only		↑	↑
Ellett et al. (74)	Australia	RCT	Some	41	Women undergoing planned laparoscopy for pelvic pain	Immediately after intervention (K), 6 weeks later (K), post-intervention (A)	Animation + Usual care vs. Usual care	↑ ↔	↔	—
Friedman et al. (95)	Israel	RCT	High	182	Women who underwent at term Induction of labour (IOL)	Post-intervention (A)	Video animation+ Standard counselling vs. Standard counselling		↑	
Gois et al. (94)	Australia	RCT	Some	124	Patients undergoing clinically indicated percutaneous kidney biopsies	Post-intervention (K, A)	Video-assisted e-consent vs. Usual consent	↑	↔	
Hermann (15)	Austria	RCT	High	80	Patients undergoing thyroid surgery	Post-intervention (K, A)	Animation vs. Written text	↔	↖	—
Homans et al. (24)	Netherlands	RCT	High	46	Participants who were eligible for cochlear implantation	Immediately after intervention, subjective & objective scores (K), Immediately after intervention (A)	3D video animations+ Standard selection process vs. Standard selection process	↑ ↔	↔	
Hong et al. (16)	Korea	RCT	High	150	Patients about to undergo CT scan	Post-intervention (K, A)	Animation vs. Usual care	↔	↑	—
Kakinuma et al. (36)	Japan	RCT	High	211	Patients about to undergo surgery for cancer	Post-intervention (K)	Animation + Usual care vs. Usual care	↑	—	—
Lattuca et al. (18)	France	RCT	Low	843	Patients undergoing coronary angiography	Post-intervention (K, A)	Animation + Usual care vs. Usual care	↑	↑	—
Lin et al. (29)	Taiwan	RCT	Low	142	Adults in Emergency Department due to have acute debridement surgery	Post-intervention (K, A)	Animation + Usual care vs. Usual care	↑	↑	—
Lv et al. (49)	China	RCT	Some	204	Caregivers whose children underwent neurosurgical procedures	Post-intervention (K)	Animation assisted education + Face-to-face oral education vs. Face-to-face oral education	↑		
Mayilvaganan et al. (37)	India	RCT	High	60	Patients undergoing thyroid surgery	Post-intervention (A)	Animation vs. 3D model	—	↔	—
							Animation vs. Static image	—	↔	—
Mednick et al. (67)	Canada	RCT	High	52	Patients undergoing an initial IVFA investigation	Post-intervention (K, A)	Animation vs. Usual care	↑	↔	—
Mhalu et al. (63)	Tanzania	RCT	Low	200	Patients at risk of pulmonary tuberculosis	Post-intervention (B)	Animation vs. Usual care	—	—	↑
Miao et al. (77)	Australia	RCT	Some	102	Patients referred for Mohs micrographic surgery	Post-intervention (K, A)	Animation vs. Usual care	↑	↔	—
Mladenovski and Kieser (75)	New Zealand	RCT	High	30	Patients referred for dental surgery	Post-intervention (K, A)	Animation vs. Leaflet	↔	↖	—
Mofrad Babapour et al. (87)	Netherlands	RCT (3 arms)	Some	209	Patients attending a memory clinic	T2 screening day (K), T3 end of screening day (K, A)	Animation viewing at home + Usual care vs. Usual care	↑ ↑	↔	—
							Animation viewing in clinic + Usual care vs. Usual care	↑ ↑	↔	—
Molher et al. (78)	France	RCT	High	69	Adults due to have planned surgery for benign parotid tumour	Post-intervention (K)	Animation + Written + Usual care vs. Written + Usual care	↑	—	—
Moore et al. (51)	USA	RCT	High	120	Adult patients undergoing Bravo placement for reflux	Post-intervention (K, A)	Written + Video instructions vs. Video instructions	↑	↑	↑
Pallett et al. (79)	USA	RCT	High	120	Women undergoing planned hysterectomy (for benign condition)	Immediately post-intervention (K, A), Day of surgery (K), 6 weeks post-surgery (K)	Animation + Usual care vs. Usual care	↑ ↑ ↔	↔	—
Platto et al. (28)	USA	RCT	High	45	Patients awaiting skin surgery	Post-intervention (A)	Animation + Usual care vs. Usual care	—	↔	—
Reynolds-Wright et al. (62)	UK	RCT and Quasi RCT	Some	172	Gynaecological patients with confirmed gestation	Post-intervention (K, A)	Animation vs. Usual care	↑	↖	—
Roy et al. (50)	Canada	RCT	Some	142	Children undergoing adenotonsillectomy	Post-intervention (A, B)	Animated audiovisual + Clinician led teaching vs. Pamphlet + Clinician led teaching	—	↔	↔
Sahebalam et al. (61)	Iran	RCT	Some	50	Primary school children referred for dental surgery	Post-intervention; 1 week later (B)	Animation vs. Tell-show-do	—	—	↑ ↑
Sariturk et al. (80)	Turkey	RCT	Some	82	Patients awaiting stem cell transplantation	Post-intervention (A)	Animation + Written + Usual care vs. Written + Usual care	—	↔	—
Shi et al. (33)	China	RCT	Some	226	Patients with atrial fibrillation (AF) undergoing atrial fibrillation catheter ablation	3 months post-intervention (A, B)	Animation + Usual care vs. Usual care		↑	↑
Shqaidef et al. (19)	Jordan	RCT	Some	64	Adolescents undergoing first orthodontic treatment	1 year post-intervention (K)	Animation vs. Leaflet	↔	—	—
Tipotsch-Maca et al. (20)	Austria	RCT	Some	123	Patients awaiting for cataract surgery	Post-intervention (K, A)	Animation + Brochure + Usual care vs. Brochure + Usual care	↖	↔	—
Tou et al. (30)	Australia	RCT	High	31	Patients undergoing bowel surgery	Post-intervention (k); day of surgery (K); 1 day later (K)	Animation + Information sheet vs. Info sheet	↔	—	—
Tucker et al. (72)	USA	RCT	Some	80	Adults due to have planned endometrial surgery for cancer staging	Pre-op (K); day of surgery (K); post -op (K, A, B)	Animation + Usual care vs. Usual care	↔ ↔ ↔	↑	®
Turkdogan et al. (81)	Canada	RCT	Some	121	Adults due to have planned head and neck surgery	Post-intervention (A)	Animation + Usual care vs. Usual care	—	↑	—
Winter et al. (38)	Australia	RCT	Low	92	Patients with acute renal colic	Post-intervention (K, A)	Animation vs. Usual care	↑	↔	—
Yap et al. (70)	Singapore	RCT (3:1)	High	332	Patients undergoing coronary angiography	Post-intervention (K)	Animation + Usual care vs. Usual care	↑	—	—

Key: ↑ favours animation, ↖ some positive results with animation, ↔ no difference between groups, ↓favours control, K = Knowledge, A = Attitude & Cognitions, B = Behaviour, IVFA =  Intravenous Fluorescein Angiography.

**Table 2:** Category 2 findings (management of long-term conditions).

Author, year	Country	Study design	ROB	Sample size	Participants	Timepoints	Interventions vs. control	Knowledge	Attitude	Behaviour
Akca Sumengen and Ocakci (41)	Turkey	RCT	Some	74	Children with chronic allergic asthma	1 month post-intervention (A, B), 4 months post-intervention (A)	Animation + Booklet+ Usual care vs. Usual care	—	↑ ↑	↑
Baker (82)	USA	RCT	Some	100	Patients undergoing testing for chronic constipation	Post-intervention (K)	Animation vs. Pamphlet	↔	—	—
Calderon et al. (59)	USA	RCT	High	240	Latino/Hispanic patients with Type 2 Diabetes	Post-intervention (K)	Animation vs. Text	↑	—	—
Chakravarthy et al. (69)	USA	RCT	Some	52	Patients prescribed opioids in Emergency Departments	Post-intervention (K)	Animation + Usual care vs. Usual care	↑	—	—
Cleeran et al. (21)	Belgium	RCT	Some	67	Patients with periodontitis	Post-test (K), 2 weeks follow up (K)	3D animation vs. Real-time sketches	↑ ↑	—	—
Diniz et al. (58)	Brazil	RCT (3 arms)	High	159	Adults with non-specific low back pain attending outpatient physiotherapy clinics	Post-intervention (K, A)	Animation vs. Infographic	®	↔	—
							Animation vs. Written information	®	↔	—
Di Pietro et al. (22)	Italy	RCT	High	144	Adult patients with atrial fibrillation (AF) or deep vein thrombosis (DVT), being discharged home from emergency department	48 h post-discharge (K, A, B)	Animation + Usual care vs. Usual care	↖	↔	↔
Flynn et al. (83)	USA	RCT	Low	120	Pregnant women with risk of pre-term birth	Time 1(K, A), Time2 (K, A), Time 3 post-intervention (K, A)	Animation + Text message vs. Weblinks + Text message	↑ ↑ ↑	↑ ↑ ↑	—
Gagne et al. (84)	Canada	RCT	Some	60	Adults with atrial fibrillation	T1 (baseline), T2 (immediately after); T3 (1 month post); T4 (3 months post); T2-T1 change (K), T3-T2 change (K), T3-T1 change (A), T4-T3 change (K, A)	Animation + Face to face vs. Face to face	↑ ↔ ↔	↔ ↔	—
Glanz 2024 (48)	USA	RCT	Low	1004	Patients receiving long-term opioid therapy (LTOT)	4 months and 8 months post-intervention (K, B)	Animation + Usual care vs. Usual care	↑ ↑		↔ ↔
Indradat (57)	Thailand	RCT	High	80	Children with allergic rhinitis	1st viewing post intervention (B), 2nd viewing post intervention (B)	Animation vs. Oral teaching	—	—	↑ ↑
Jones et al. (85)	New Zealand	RCT	High	70	Patients with acute coronary syndrome	Post-intervention (K, A, B)	Animation + Usual care vs. Usual care	↖	↖	↖
Jones et al. (35)	New Zealand	RCT (3 arms)	Some	96	Patients after surgery	Post-intervention (A)	Animation + Usual care vs. Active control without animation + Usual care	—	↔	—
							Animation + Usual care vs. Usual care	—	↔	—
Kaewin et al. (40)	Thailand	Quasi RCT	High	42	Children with epilepsy	8 weeks post-intervention (B)	Animation vs. Usual care	—	—	↑
Kayler et al. (56)	USA	RCT	Some	80	Kidney donation, patients	Post-intervention (K, A, B)	Animation + Usual care vs. Usual care	↑	↔	↖
Kocaaslan et al. (98)	Turkey	RCT	Some	93	Children with asthma, aged 7–11 years	1 month and 3 months post-intervention (A, B)	Animation vs. Usual care		↔ ↑	↑ ↑
Li et al. (86)	China	RCT	Some	80	Patients with lung cancer, preparing for surgery	Post-intervention (K, B)	Animation vs. Face to face	↑	—	↔
McIntyre et al. (68)	Australia	RCT	Low	208	Patients with Atrial fibrillation (AF) attending outpatient cardiology clinics	2 days and 90 days post-intervention (K, A)	Animations + Standard care vs. Standard care	↔ ↑	↔	
Nana et al. (39)	Cameroon	RCT	High	110	Adults with hypertension	Post-intervention (B)	Animation + Usual care vs. Usual care	—	—	↔
Saengrow et al. (55)	Thailand	RCT	High	214	Children's use of anti-epileptics	Immediately post-intervention (K, B)	Animation + Advice vs. Advice only	↑	—	↑
Schroeder et al. (54)	USA	RCT	High	98	Adults with urinary incontinence	Immediately after education (K, A), 6–8 weeks later (K, A)	Animation vs. Face to face	↔ ↔	↔ ↔	—
Sommer et al. (88)	Switzerland	RCT	Some	43	Adults with keratoconus	Immediately after the consultation (K)	Animation+ Face to face vs. Face to face	↑	—	—
Wonggom et al. (65)	Australia	RCT	High	36	Patients with heart failure	30 days post-intervention (K, A, B), 90 days post-intervention (K, A, B)	Animation + Usual care vs. Usual care	↔ ↑	↔ ↔	↔ ↔
Ye et al. (23)	China	RCT	High	66	Adults following gastrointestinal surgery	T1 (pre-op after intervention), T2 (1 week post-surgery), T3 (2 weeks post-surgery), T4 (1 month post-surgery), T5 (3 months post-surgery) (all B)	Animation + Verbal information vs. Verbal information only	—	—	↑ ↑ ↑ ↑ ↔

Key: ↑ favours animation, ↖ some positive results with animation, ↔ no difference between groups, ↓ favours control, K = Knowledge, A = Attitude & Cognitions, B = Behaviour.

**Table 3:** Category 3 findings (topics related to public health, health promotion, illness prevention, or screening).

Author, year	Country	Study design	ROB	Sample size	Participants	Timepoints	Interventions vs. control	Knowledge	Attitude	Behaviour
Acharya (13)	UK	RCT	Low	5,933	Women invited for mammography. (Timed appointments)	3 months post-intervention (B)	Animation+ Behavioural SMS vs. Behavioural SMS	—	—	↔
Acharya (13)	UK	RCT	Low	16,366	Women invited for mammography. (Open appointment strategy)	3 months post-intervention (B)	Animation+ Behavioural SMS vs. Behavioural SMS	—	—	↔
Adam et al. (42)	South Africa	Cluster RCT	High	1,502	Mothers of infants; focus on breastfeeding	1 month (K,B), 5 months (K,B)	Animation + Usual care vs. Usual care	↑ ↔	—	↔ ↔
Basir et al. (97)	USA	RCT	Some	50	Fathers living with partners who have high-risk pregnancies	25, 30, 34 weeks post-intervention (K,A)	Animated videos vs. Webpage link to American College of Obstetricians and Gynaecologists	↔ ↔ ↔	↑ ↔ ↔	
Bayraktar Nahir et al. (46)	Turkey	RCT (3 arms)	High	490	Primary school students aged 10–12 years	Post-intervention (K)	Animation vs. Verbal explanation	↔		
							Animation vs. Peer-led reels	↑		
Boontor et al. (92)	Thailand	RCT	Low	202	Postpartum women aged 18 years and above	Immediate and 12 weeks post-intervention (B)	Animated video + Standard care vs. Standard care			↑↔
Bukkhunthod et al. (53)	Thailand	Cluster RCT	Some	80	School children aged 9–12 years	Post-intervention (K)	Animation vs. Traditional programmes	↑	—	—
Burapasikarin et al. (89)	Thailand	RCT	Low	270	Postpartum women	6–8 weeks post-intervention (B)	Animation + Usual care vs. Usual care	—	—	↑
Choa et al. (52)	South Korea	Cluster RCT	Some	85	Hospital employees	Post-intervention (B)	Animation assisted CPR vs. Dispatcher assisted CPR	—	—	↖
Cooper et al. (96)	UK	RCT	High	52	Pregnant women between 20 and 24 weeks’ gestation	6 weeks post-intervention (B)	Digital health intervention (DHI)+ Standard care vs. Standard care			↔
Ehsani et al. (17)	Iran	RCT	High	371	Adolescents undergoing dental treatment	Post-intervention (K, B)	Animation vs. In-person information	↑	—	↔
Gafni-Amsalem et al. (43)	Israel	RCT	High	304	Couples and individuals considering genetic testing	Post-intervention (K, A)	Animation vs. Booklet	↔	↔	—
Housten et al. (34)	USA	RCT (3 arms)	Some	187	People using a community food bank or attending the Houston Cancer Prevention Centre	Post-intervention (K)	Animation vs. Video with static image	↔	—	—
							Animation vs. Audio booklet	↔	—	—
Kasthuripriya et al. (47)	India	RCT (3 arms)	Low	90	Orphaned adolescents, aged 12–15 years, with plaque-induced gingivitis	3 months and 6 months post-intervention (K, A)	Cartoon animation vs. Pamphlet	↑ ↑	↔ ↑	
							Cartoon animation vs. Caregiver-supervised training	↔ ↔	↑ ↔	
Kumar et al. (44)	India	RCT	Low	80	Adolescents with hearing or speech impairments	16 weeks post- intervention (K, B)	Animation + In-person demonstration vs. In-person sign language + In-person demonstration	↑	—	↖
Leiner et al. (5)	USA	RCT	High	192	Parents of children receiving polio vaccines	Post-intervention (K)	Animation vs. Printed information	↑	—	—
Meppelink et al. (32)	Netherlands	RCT	Some	231	Participants 55 + with either low or high health literacy	Post-intervention (K)	Animation vs. Static illustration	↖	—	—
Nintao et al. (45)	Thailand	RCT	Low	176	Pregnant women (gestational age 14 weeks or less)	Immediately post- intervention (K, A)	Animation + Usual care vs. Usual care	↑	↔	—
Rakhmilla et al. (90)	Indonesia	Quasi-RCT (3 arms)	High	180	Senior High School students	Post-intervention (K)	Animation vs. Peer education	↔	—	—
							Animation vs. Conventional lecture education	↑	—	—
Romantika et al. (31)	Indonesia	Quasi-RCT	High	120	Mothers of children aged 4–7 years	Post-intervention (K, A)	Animation vs. Leaflet	↑	↑	—
Ruparel et al. (91)	UK	RCT	High	246	Smokers/former smokers	Post-intervention (K, A)	Animation + Booklet vs. Booklet	↑	↑	—
Schnellinger et al. (4)	USA	RCT	High	162	Parents of paediatric patients	1–2 h post-intervention (K, A), 4 weeks post-intervention (K)	Animation vs. Pamphlet	↔ ↑	↔	—
Tongpeth et al. (66)	Australia	RCT	Low	70	Adults who had an MI (heart attack)	1 month after discharge (K, A, B), 6 months after discharge (K, A, B)	Animation + Usual care vs. Usual care	↑ ↑	↑ ↑	↑ ↑
Yuen and Mak (60)	Hong Kong	RCT	Low	137	General public attitudes to mental illness	Immediately after intervention (A), 1 week after intervention (A)	Animation vs. Text	—	↔ ↔	—

Key: ↑ favours animation, ↖ some positive results with animation, ↔ no difference between groups, ↓favours control, K = Knowledge, A = Attitude & Cognition, B = Behaviour.

#### 3.1.2 Access to animations

Participants' access to animations was reported in 60 trials. In 33 trials participants viewed the animation just once (4, 15, 16, 18, 19, 22, 23, 31, 34, 40–46, 54–56, 58, 60–64, 72, 76, 79, 80, 82, 84, 88, 89) and one study allowed participants to choose to view it once or twice (91). In two trials participants viewed the animation twice (86) or three times (53).

In fourteen trials access to the animation was either unlimited (24, 39, 48, 49, 51, 65, 68, 78, 85, 97) or unlimited over a specific time frame, such as four weeks (17), three months (13) or six months (66). In three trials viewing was unlimited during a clinic visit (21, 50) or within a 30 min period during a clinic visit (36). In another four trials participants viewed the animation during a clinic visit and were allowed to pause the video, rewind it, and ask questions (25–27, 94).

In one study patients could watch the animation once if they were in the clinic (clinic viewing group) or had unlimited viewing if they were at home (home viewing group), according to allocation (87). The videos were viewed multiple times in one study until participants were able to demonstrate competence or a thorough understanding of the topic (57).

In 28 trials the level of access was not specified (5, 20, 28–30, 32, 33, 35, 37, 38, 47, 52, 59, 67, 69–71, 73–75, 77, 83, 90, 92, 93, 95, 96, 98). Out of the 88 included trials, 30 publications provided a link to the tested animation, while 58 either did not provide a link or it was not working.

#### 3.1.3 Comparators and alternatives to animations

In 49 trials animations were included as a supplementary intervention:

- Standard care (18, 22–24, 29, 33, 35, 39, 41, 42, 45, 48, 65, 66, 68, 69, 72, 74, 76, 79–81, 85, 87, 89, 92, 95, 96);
- Spoken information plus brochure (20, 64);
- Consultation with a doctor (i.e., spoken information) with or without written information (28, 36, 54, 55, 78, 88);
- Verbal consent (71);
- Printed booklet (30, 91);
- Written and spoken information (25–27, 51, 93);
- Nurse education audio-recording (56);
- Face to face education (49, 84);
- Behavioural digital text message (13).

In 38 trials the animation was given as an alternative intervention:

- Spoken information (16, 17, 46, 54, 77, 86);
- Usual care (40, 62, 63, 70, 94, 98);
- Static images (21, 32);
- Either diagram or 3D model, by allocation (37);
- Written information (printed or digital) (4, 5, 15, 19, 31, 43, 47, 50, 60, 73, 75, 82);
- A combination of booklet, poster and spoken information (53);
- Live instructions by phone (52);
- The Tell-Show-Do technique (61);
- Website links (83, 97);
- In-person sign language instructions (44);
- Audio-booklet or static images, by allocation (34);
- Infographic or written information, by allocation (58);
- Peer education or conventional lecture, by allocation (90);
- Short film or standard care, by allocation (76);
- Verbal consent following spoken information (38).

In one trial (67) the animation was offered as an alternative to the standard physician-patient consent conversation in one trial arm, and an addition to it in another trial arm.

#### 3.1.4 Outcome measures

Knowledge was the most commonly reported outcome, being included in 60 trials (4, 5, 15–22, 24, 29–32, 34, 36, 38, 42–49, 51, 53–56, 58, 59, 62, 64–75, 77–79, 82–88, 90, 91, 94, 97).

Attitudes and cognitions were reported in 53 trials, including information satisfaction (15, 18, 20, 22, 24–27, 29, 30, 38, 43, 45, 50, 51, 62, 64, 67, 68, 71–75, 77, 79–81, 87, 93–95); self-efficacy (56); illness perceptions (85); quality of recovery (35); quality of life (41, 54, 84); information satisfaction, unmet information needs (37); information satisfaction, perceived familiarity with topic (16); desire for information (28); self-care confidence (65); attitude to information (31, 47, 58, 60, 66); subjective knowledge, decisional certainty (91), preparation for decision making (83); and information satisfaction, having learned from information (4, 16, 62, 76).

Thirty one trials reported behaviour outcomes, including willingness to consent to the medical procedure (25–27, 56); physical activity (86); contraception use (89, 92); return to work, physical activity and medication adherence (85); time taken to initiate cardio-pulmonary resuscitation (52); self-care behaviours (40, 65); quality of sputum sample (63); patient co-operation (61), visit length (72), school absenteeism (41); anticoagulant initiation rate (22); competence in using inhaler (57); medication adherence or reduction in rescue medication use (33, 39, 55, 68, 98); completion of training content (23); attendance at screening (13); breastfeeding behaviour (42); teeth cleaning method (17, 44); making a post-heart attack action plan (66); resource utilisation (50); and opioid risk behaviour (48).

Only seven trials reported all three categories of outcome (i.e., knowledge; attitudes and cognitions; behaviour) (22, 51, 56, 65, 66, 72, 85).

#### 3.1.5 Timing of outcome assessment

The timing of outcome assessments was highly variable, ranging from immediately after the delivery of the intervention to 1 year afterwards (see Tables 1–3).

### 3.2 Settings

As with the 2022 version of the systematic review, we categorised trials into three groups based on the intended purpose or context of the information:

Category 1: Explaining medical or surgical procedures (40 trials);
Category 2: Management of health conditions (24 trials);
Category 3: Topics related to public health, health promotion, illness prevention or healthy person screening (24 trials).

#### 3.2.1 Category 1: explaining medical or surgical procedures (40 trials)

**Figure 2:**
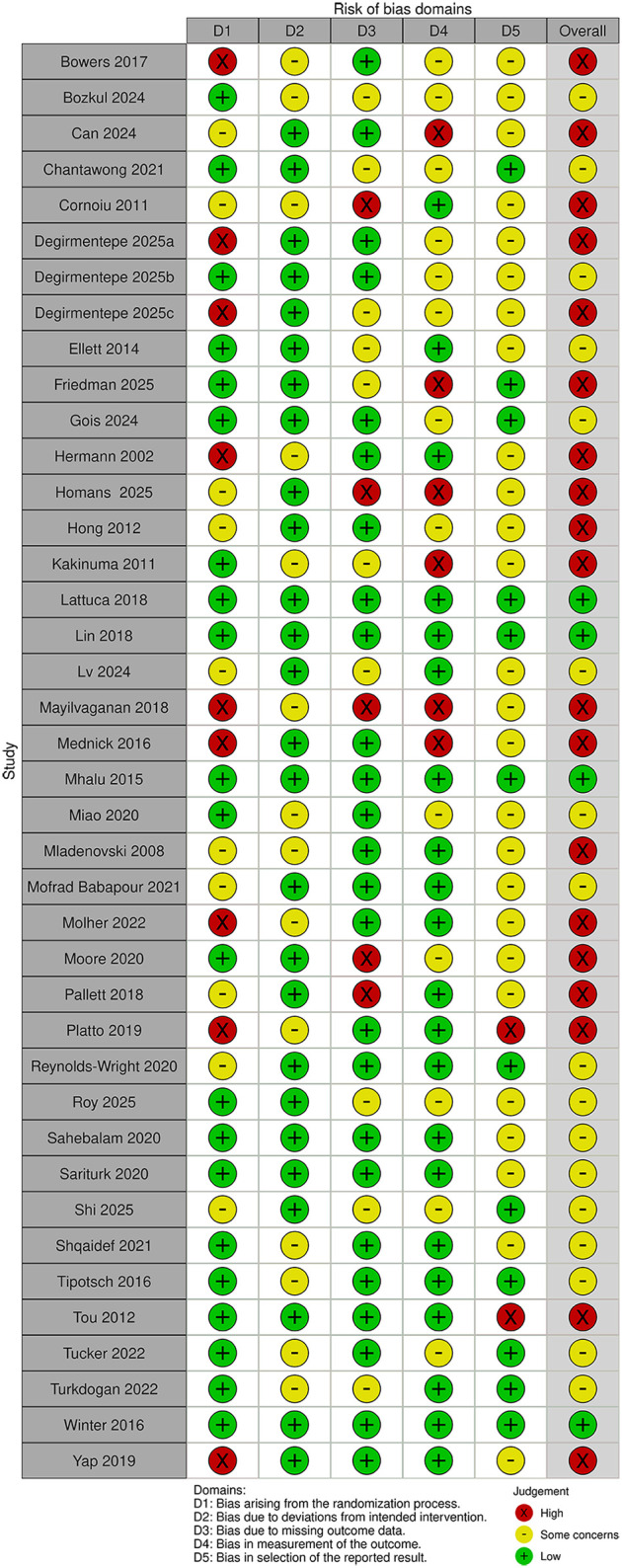
Risk of bias in the Category 1 studies.

Figure 2 and Table 1 summarise the risk of bias judgements and findings across the trials in category 1 (40 trials, total *n* = 5,388, sample range 30–843) (15, 16, 18–20, 24–30, 33, 36–38, 49–51, 61–64, 67, 70–81, 87, 93–95).

Nineteen of the 40 trials were assessed as having a high risk of bias, most commonly due to the randomisation process. The other trials were rated as at low risk of bias (four trials) (18, 29, 38, 63) or having “some concerns” (17 trials), due to higher dropout rate, lack of protocol registration, or unblinded outcome assessment (19, 20, 25, 33, 49, 50, 61, 62, 64, 72, 74, 76, 77, 80, 81, 87, 94).

##### 3.2.1.1 Effects on knowledge

Knowledge was evaluated in 26 Category 1 trials, and the provision of an animation resulted in positive outcomes in fifteen of those trials (18, 29, 36, 38, 49, 51, 62, 67, 70, 71, 73, 77, 78, 87, 94). Two trials reported some positive results (20, 24), while three other trials showed mixed results at different time points (i.e., favoured animation immediately after intervention but not at 6 weeks follow up) (74, 79) or across the arms in a three-arm study (i.e., favoured animation in one comparison, with no difference in the other comparison) (64). Six trials showed no statistically significant differences between the intervention and control groups (15, 16, 19, 30, 72, 75). It is notable that knowledge outcomes favoured the animation in nearly all trials (11 out of 12) when the comparator was standard care or spoken information, and favoured the animation in five out of seven trials when the comparator was written information, such as a brochure, written text, leaflet or pamphlet.

No Category 1 trial reported better knowledge outcomes in the control group (See Supplementary Materials: Supplementary Table 1 for a detailed summary).

##### 3.2.1.2 Effects on attitudes and cognitions

Attitudes and cognitions were assessed in 32 trials in Category 1 and 13 trials reported statistically significant differences favouring the animation (16, 18, 25–27, 29, 33, 51, 71, 72, 81, 93, 95). In two of the three-armed trials, the results were mixed; for example, one comparison showed a preference for animation, while another comparison showed either no difference or a preference for the control intervention (73, 76). Four trials showed some benefits of animation (i.e., outcomes favoured animation in some items or sub-scores, but no differences between arms with the remainder) (15, 37, 62, 75). Twelve trials reported no statistically significant differences between groups (20, 24, 28, 38, 50, 64, 67, 74, 77, 79, 80, 87, 94).

No Category 1 trial reported better attitudes and cognitions outcomes in the control group.

##### 3.2.1.3 Effects on behaviours

Behaviours were evaluated in nine Category 1 trials (25–27, 33, 50, 51, 61, 63, 72). Six of these trials showed positive results for the animations (25–27, 51, 61, 63). One trial reported no statistically significant differences between groups (50). One trial found that patients who watched the animation produced higher quality sputum samples (63), while another trial found the animation was more effective in preparing children for dental treatment (61).

One trial, which included preoperative counselling, reported that healthcare visits were significantly longer for the animation group compared to those receiving standard physician education (72).

#### 3.2.2 Category 2: management of health conditions (24 trials)

**Figure 3:**
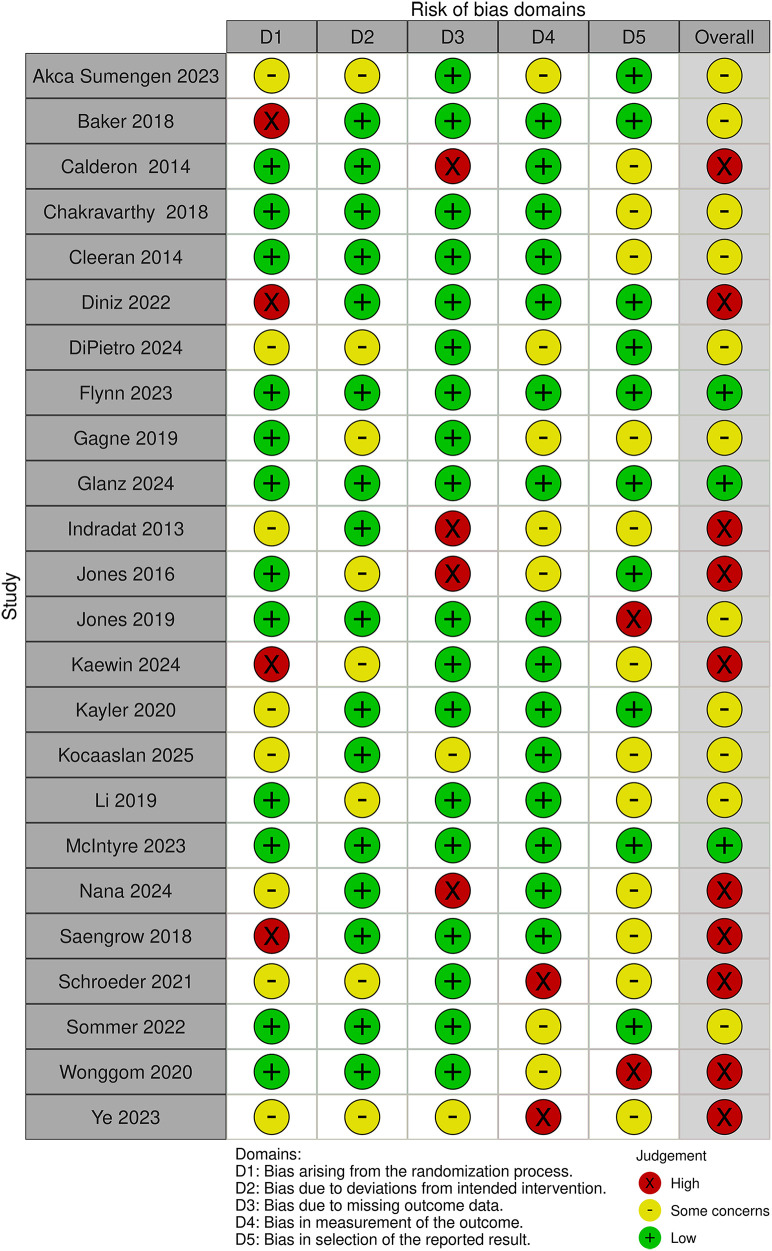
Risk of bias in the Category 2 studies.

Figure 3 and Table 2 summarise the risk of bias judgments and findings across trials in Category 2, which includes 24 trials with a total of 3,336 participants (sample range 36–1,004).

Ten trials were rated as having a high risk of bias because of issues related to the randomisation process, missing data, being underpowered (due to an inability to recruit the target sample size), or lack of blinding of outcome assessors (23, 39, 40, 54, 55, 57–59, 65, 85).

Eleven trials were rated as having “some concerns” regarding bias. This was due to a range of factors, including unclear randomisation, blinding of outcome assessors, small sample sizes, lack of protocol registration, use of unvalidated measures or self-reported outcomes (21, 22, 35, 41, 56, 69, 82, 84, 86, 88, 98).

Only three trials in this category were rated as having a low risk of bias (48, 68, 83).

##### 3.2.2.1 Effects on knowledge

Knowledge was evaluated in 17 of the 24 Category 2 trials, and the use of an animation resulted in positive outcomes in nine trials (21, 48, 55, 56, 59, 69, 83, 86, 88).

Two trials that included patients with cardiovascular disease (22, 85) reported some benefits from the animation, while three other trials reported mixed outcomes at different time points (65, 68, 84). For instance, one study showed better outcomes for animations immediately after the intervention but not 1–3 months later (84), while the other two trials reported benefits from animations at a later follow-up period (90 days) (65, 68). However, two trials showed no differences between arms (54, 82).

Notably, one trial that compared animations with infographics or written content reported better knowledge outcomes in the control group (58) (See Supplementary Materials: Supplementary Table 2 for a detailed summary).

Interestingly, two trials that tested animations on participants with low health literacy produced conflicting results. One trial found a positive effect of the animation compared to the control group (59), while the other trial showed no statistically significant differences between interventions (82).

##### 3.2.2.2 Effects on attitudes and cognitions

Attitudes and cognitions were assessed in 12 of the 24 trials. Of these, two trials showed a positive effect of animation: one focused on health promotion in children with chronic allergic asthma (41), and the other addressing birth education for pregnant women at risk of preterm birth (83). Additionally, two trials indicated some improvements from animations on aspects of outcome measures or at different time points (85, 98). In contrast, eight trials reported no significant differences between the groups (22, 35, 54, 56, 58, 65, 68, 84).

No category 2 study reported better attitudes and cognitions outcomes in the control group.

##### 3.2.2.3 Effects on behaviours

Behaviours were assessed in 13 Category 2 trials. Five trials reported statistically significant results that favoured the use of animation (40, 41, 55, 57, 98), while two trials showed some benefits from animation (e.g., favoured animation on 1 out of 4 measures (85); and favoured animation for IRD willingness only (56). One study examining the completion rates of pelvic floor muscle training after surgery found there was a preference for animation at various time points: pre-operation, one week, two weeks and one month after surgery. However, no significant differences were noted at three months post-surgery (23). In the remaining five trials, there were no statistically significant differences between the intervention and control groups regarding compliance and self-care behaviour (22, 39, 48, 65, 86).

No Category 2 trial reported better behaviour outcomes in the control group.

#### 3.2.3 Category 3: topics related to public health, health promotion, illness prevention or healthy person screening (24 trials)

**Figure 4:**
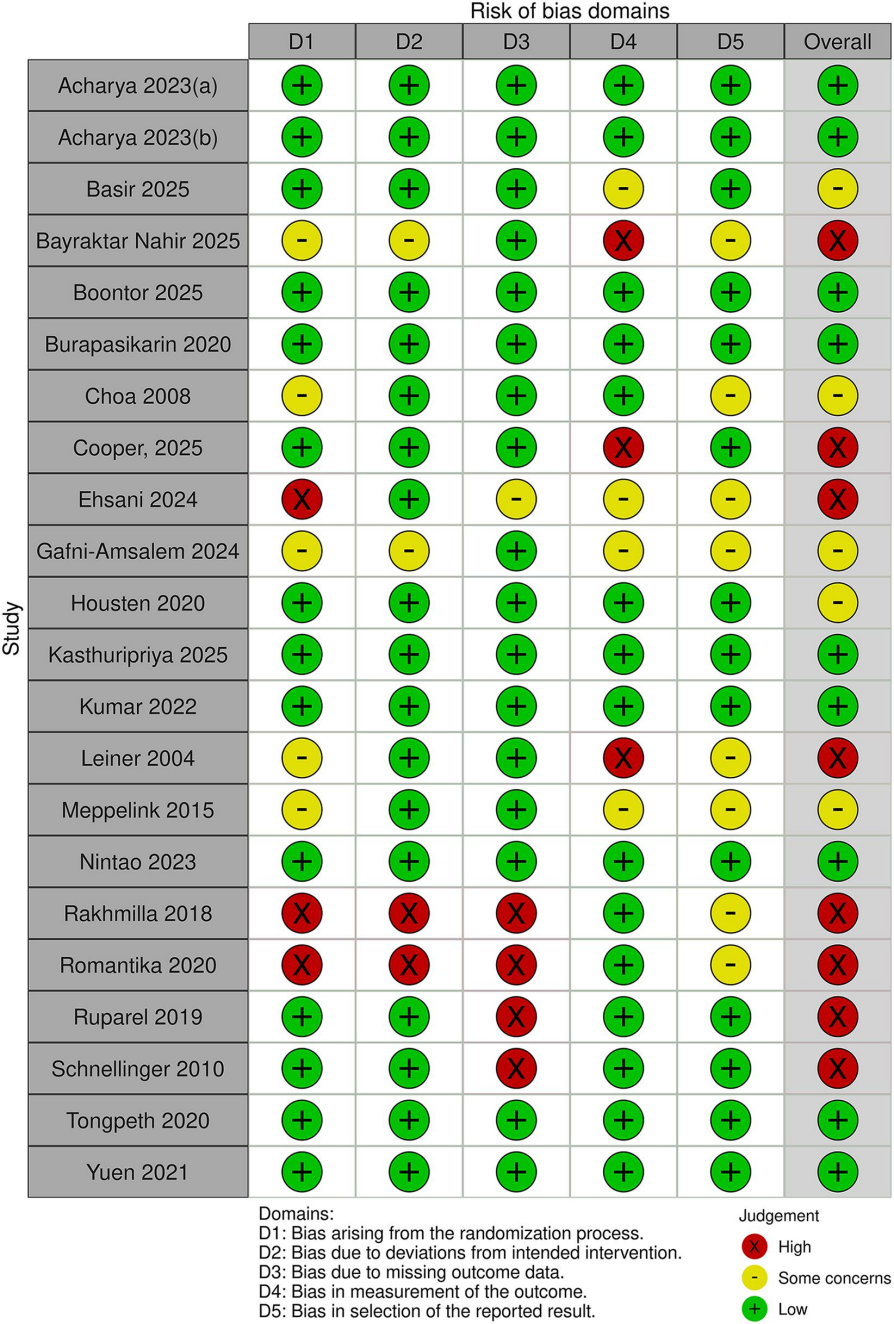
Risk of bias in the Category 3 studies.

**Figure 5:**
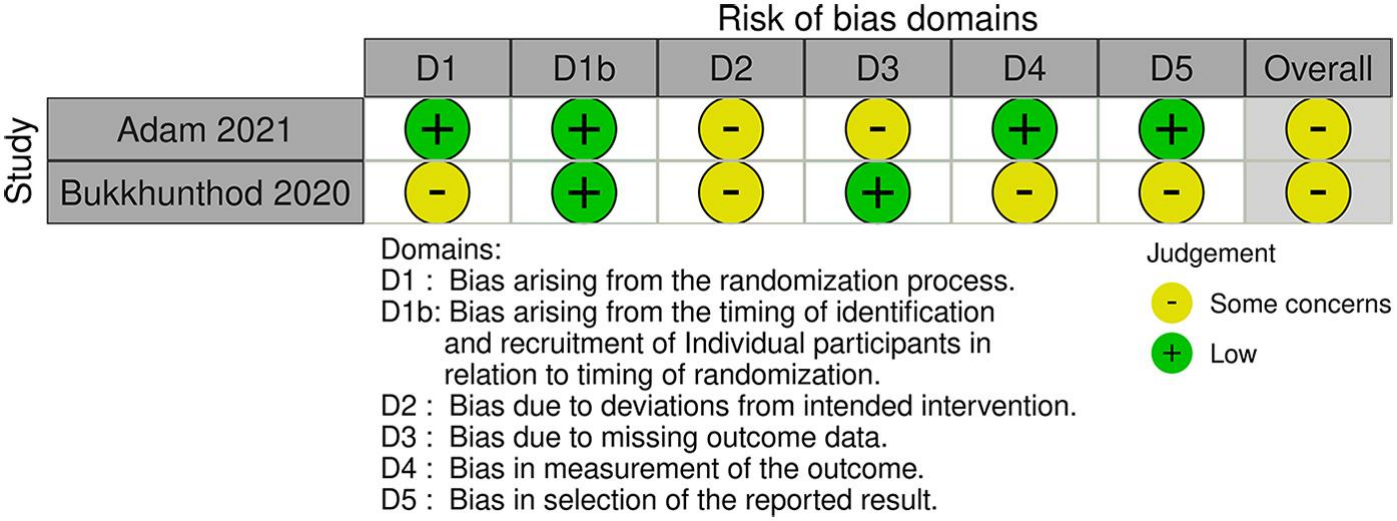
Risk of bias in the Category 3 Cluster trials.

Figures 4, 5 and Table 3 summarise the risk of bias assessments and findings across 24 Category 3 trials, involving a total of 29,038 participants (sample range 50–16,716). Out of the 24 trials eight were rated as having a high risk of bias (4, 5, 17, 31, 46, 90, 91, 96). The most common risks were the randomisation process, missing outcome data, and deviations from the intended interventions. Seven trials, including two clustered RCTs, had “some concerns” about bias due to unclear randomisation and blinding of outcome assessors, bias in measurement of the outcome or the absence of a predefined protocol or sample size calculation (32, 34, 42, 43, 52, 53, 97). Nine trials were assessed as having a low risk of bias (13, 44, 45, 47, 60, 66, 89, 92).

##### 3.2.3.1 Effects on knowledge

Knowledge was assessed in 17 of the 24 Category 3 trials and provision of an animation resulted in positive outcomes in eight (5, 17, 31, 44, 45, 53, 66, 91). One trial (32) showed some benefits from animations (i.e., only in some participant groups), while five other trials showed mixed outcomes at different time points (i.e., favoured animation at 4 weeks follow-up but not immediately after the intervention) (4, 42) or across the different arms in a three-arm trial (46, 47, 90). Three trials which compared an animation with a booklet, audio booklet or webpage link, reported no differences between arms (34, 43, 97).

No Category 3 trial reported better knowledge outcomes in the control group (see Supplementary Materials: Supplementary Table 3 for a detailed summary).

##### 3.2.3.2 Effects on attitudes and cognitions

Attitudes and cognitions were assessed in nine of the 24 Category 3 trials. Among these, three trials reported significant differences favouring animation (31, 66, 91), while two trials showed mixed outcomes at different time points (97) or across the different arms in a three-arm trial (97). The remaining four trials found no significant differences between the groups (4, 43, 45, 60). Notably, three of the four trials that showed no difference had compared an animation with written information, such as booklets, pamphlets or text (4, 43, 60).

No Category 3 trial reported better attitudes and cognition outcomes in the control group.

##### 3.2.3.3 Effects on behaviours

Behaviours and skills were assessed in 10 of the 24 Category 3 trials. Two trials, one focusing on the use of long-acting reversible contraception (LARC) in postpartum women and another on patients after myocardial infarction, reported positive results in favour of video animation (66, 89). Additionally, two trials demonstrated some benefits from using animations: one compared live CPR instructions provided by a dispatcher over the phone with video animation, and the other involved adolescents with hearing or speech impairment (44, 52). One trial showed mixed outcomes at different time points (92). However, the remaining five trials, which compared animations with behavioural text, in-person information or standard care, showed no statistically significant differences in compliance or self-care behaviours between the intervention and control groups (13, 17, 42, 96).

No Category 3 trial reported better behaviour outcomes in the control group.

#### 3.2.4 Combined results across the three categories and albatross plots

**Figure 6:**
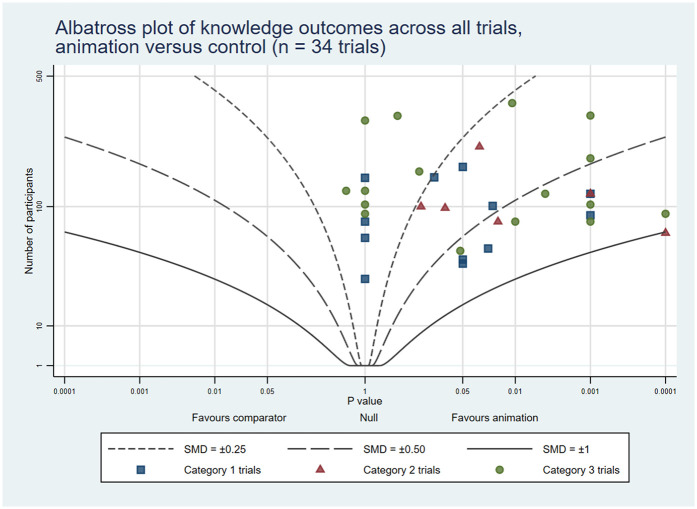
Albatross plot of knowledge outcomes across all trials Animation vs. Control.

**Figure 7:**
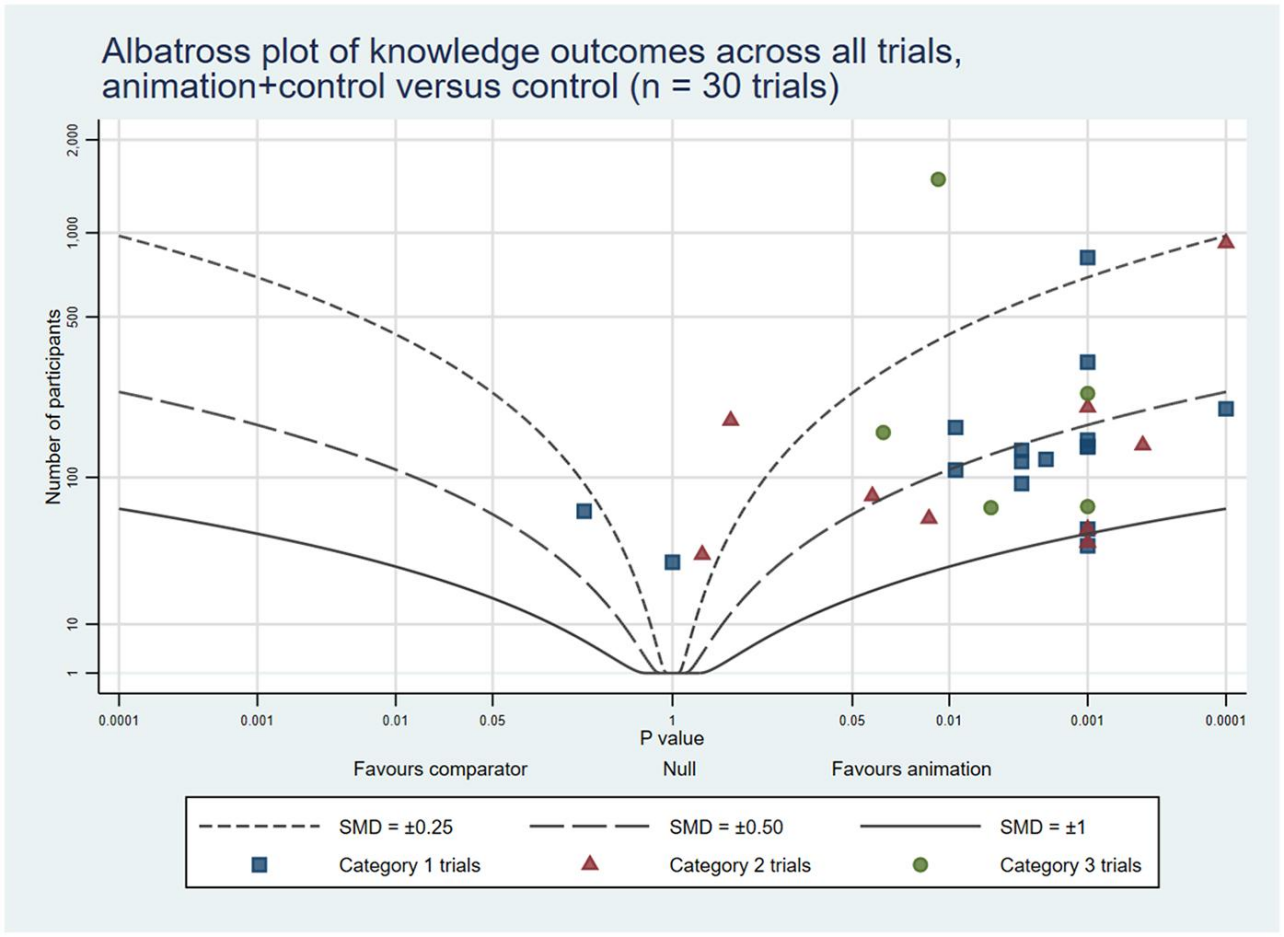
Albatross plot of knowledge outcomes across all trials Animation+Control vs. Control.

**Figure 8:**
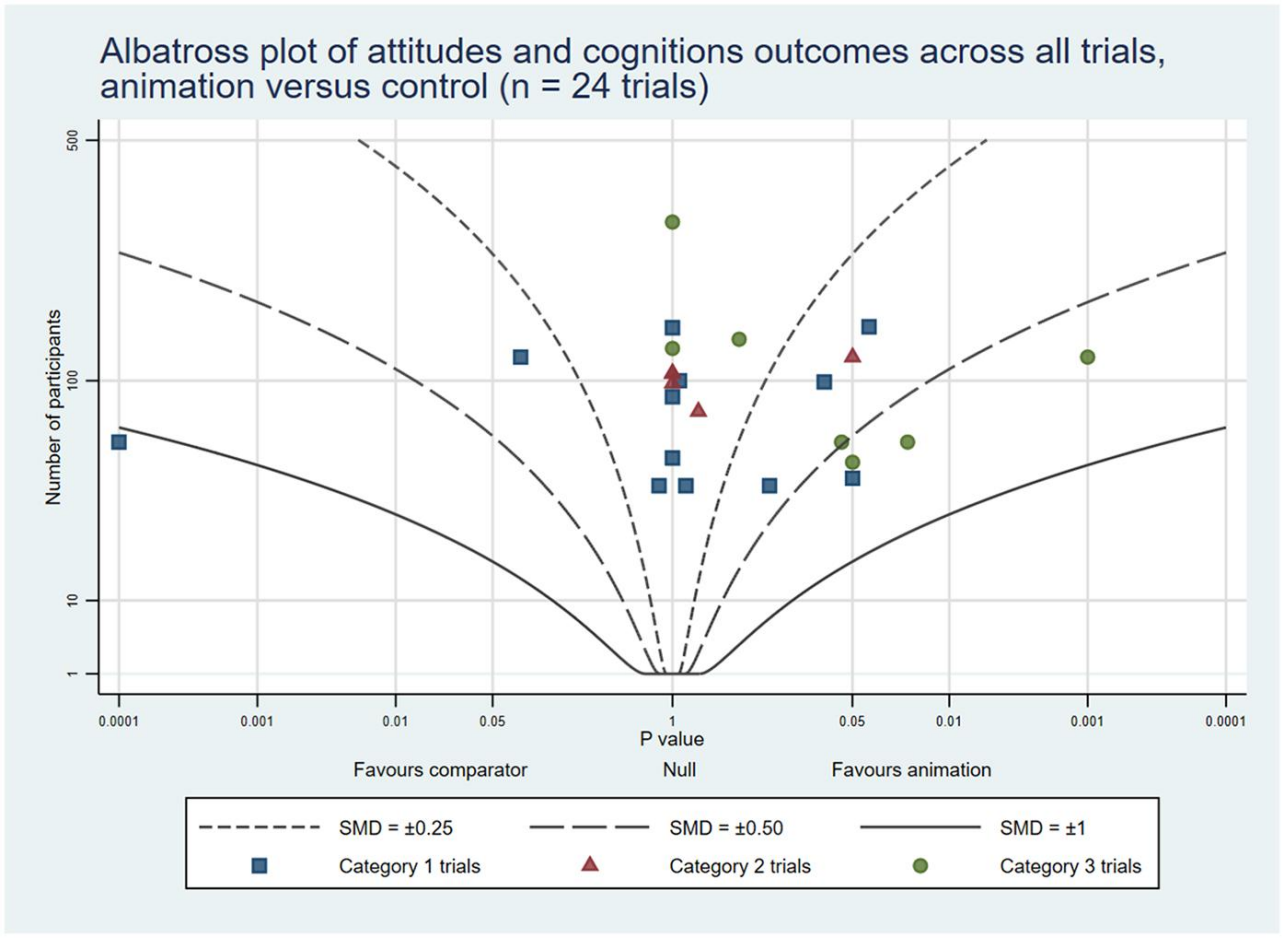
Albatross plot of attitudes and cognitions outcomes across all trials Animation vs. Control.

**Figure 9:**
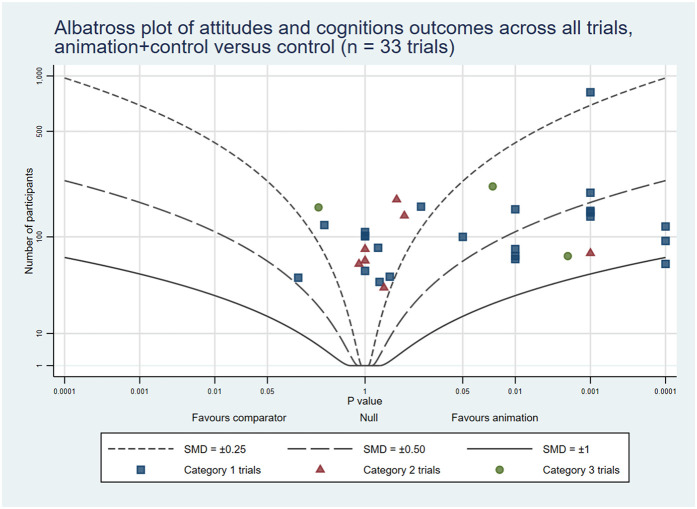
Albatross plot of attitudes and cognitions outcomes across all trials Animation+Control vs. Control.

**Figure 10:**
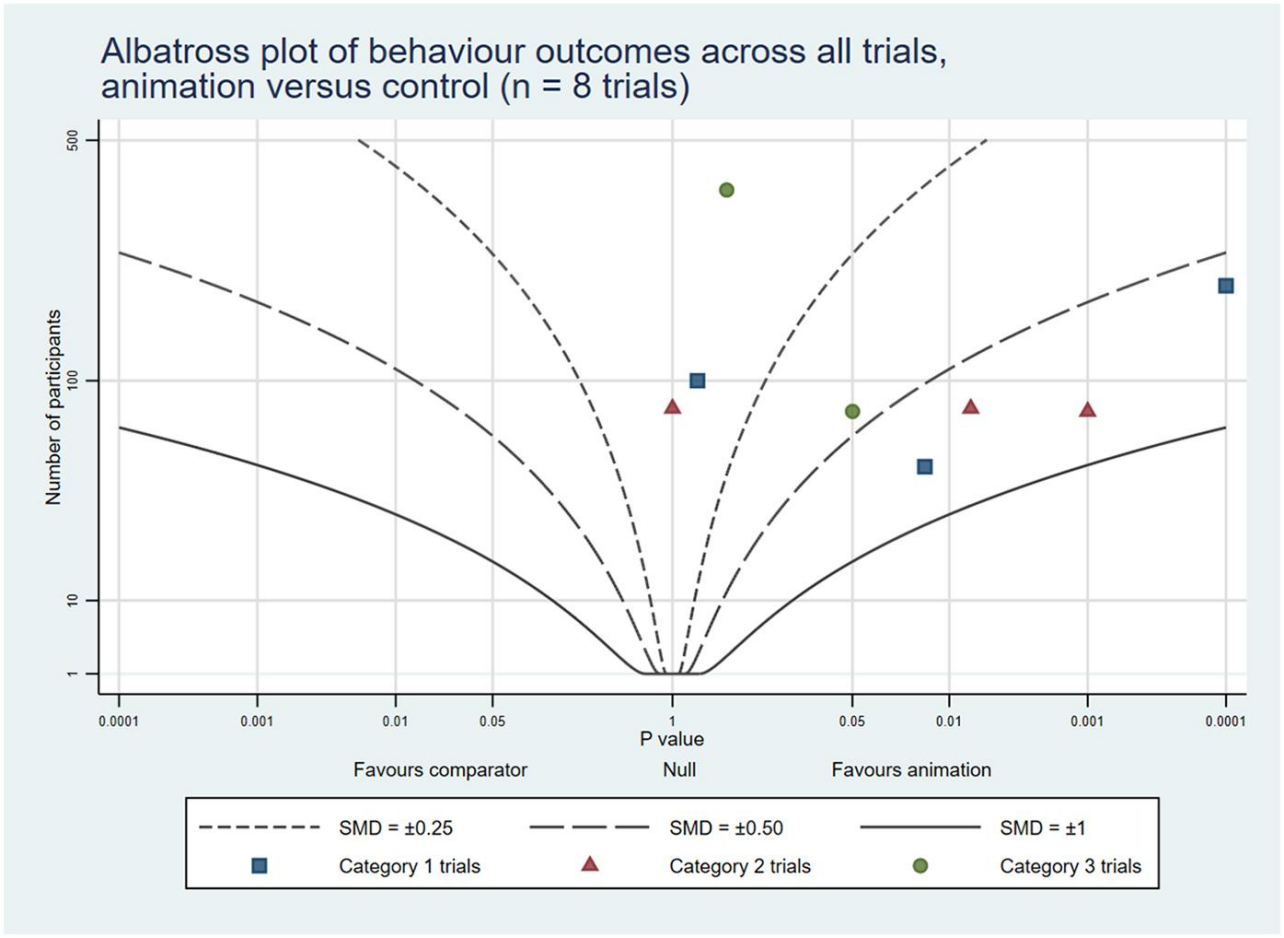
Albatross plot of behaviour outcomes across all trials Animation vs. Control.

**Figure 11:**
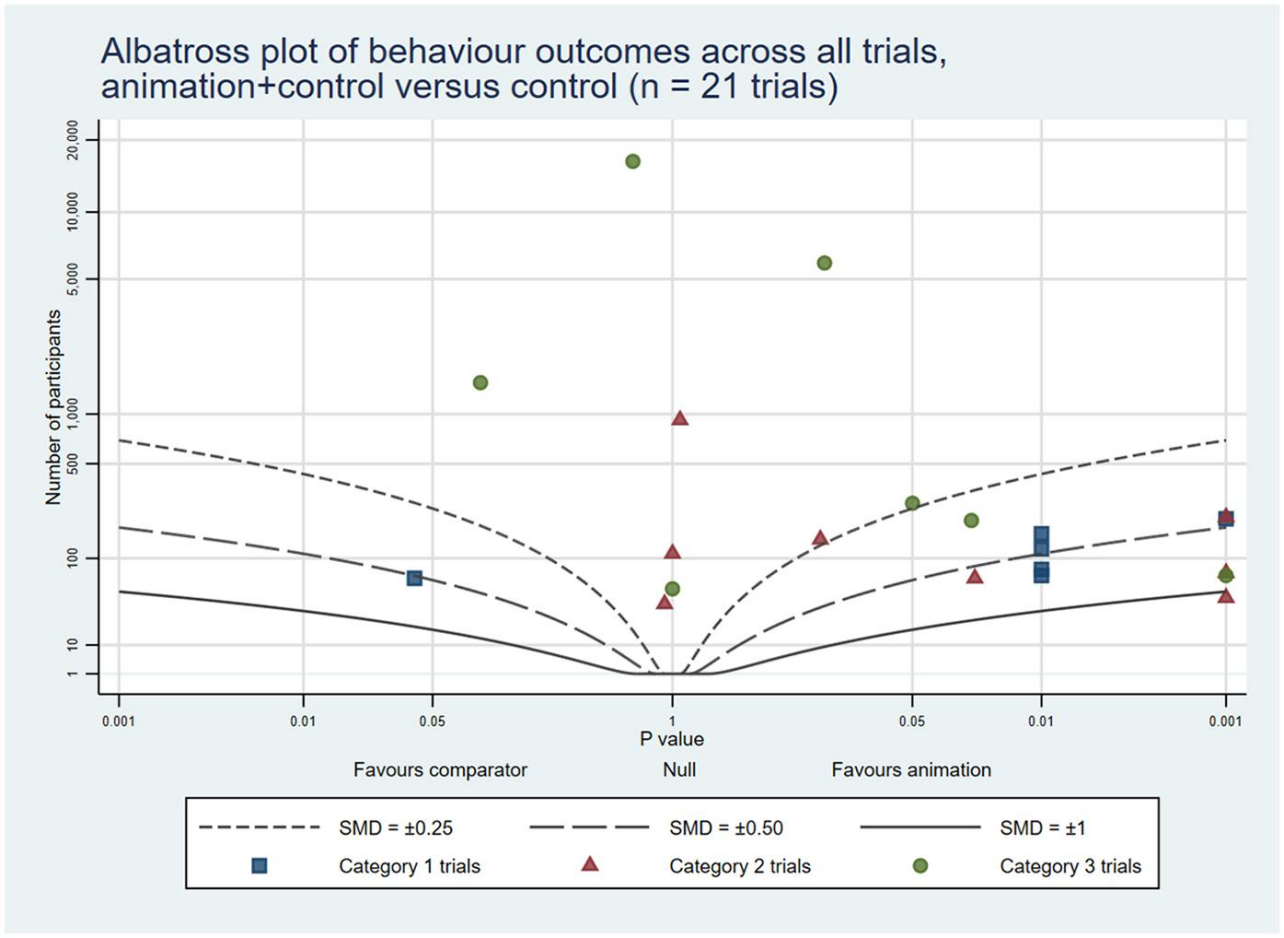
Albatross plot of behaviour outcomes across all trials Animation+Control vs. Control.

The combined rates of statistically significant results favouring animations across the three trial categories were: knowledge 32 (53%); attitudes and cognitions 18 (34%); and behaviours 14 (44%). In addition, 16 (27%), 10 (19%) and 6 (19%) trials showed some benefits associated with animations, respectively. On the other hand, 11 (18%), 25 (47%) and 11 (34%), respectively, reported no differences between the groups. Lastly, 1 (2%), 0 (0%), and 1 (3%), respectively, indicated negative outcomes related to animations across all trial categories (see Table 4). The albatross plots included 64 comparisons assessing knowledge (34 of animations as an alternative and 30 of animations as an additional format), 57 comparisons assessing attitudes and cognitions (24 as alternatives and 33 as additions) and 29 comparisons assessing behaviour (8 as alternatives and 21 as additions). The albatross plots illustrate that on all three outcome categories most trials reported positive effects of the video animations at the first post-intervention time point (see Figures 6–11).

**Table 4:** Combined results across 3 outcome categories.

Outcomes	Knowledge (*n*=60)	Attitudes and Cognitions (*n*=53)	Behaviour (*n*=32)
Positive	32 (53%)	18 (34%)	14 (44%)
Some benefit	16 (27%)	10 (19%)	6 (19%)
No difference	11 (18%)	25 (47%)	11 (34%)
Negative	1 (2%)	0 (0%)	1 (3%)

## 4 Discussion

### 4.1 Summary of findings

This updated systematic review of trials of video animations as information tools for patients and the public included 87 publications (88 trials), including 50 trials new to this update. Due to significant variation across the trials, data pooling was not possible. Most trials assessed the effect of cartoons or 3D animations. Knowledge was the outcome most often assessed, usually shortly after information delivery, and eighty percent of the trials that reported knowledge outcomes indicated a positive or somewhat positive effect of animations, especially when the comparison was standard care. Compared to knowledge outcomes, evaluations of participants' attitudes and cognitions were less common, showing benefits of animations in some trials but lacking clear benefits in others. 53% of trials measuring attitudes and cognitions outcomes showed positive effects of animations. Patient behaviour was evaluated least often, with 63% of trials reporting positive effects from animations. Across the 88 trials, only two showed significant benefits of the control intervention compared to animation (58, 72). Only three trials specifically focused on evaluating the effectiveness of animations for people with low health literacy (34, 59, 82).

### 4.2 Strengths and limitations

This is the largest systematic review of video animations in healthcare, public health and health education settings, including almost 90 trials. Several review processes were employed to minimise risk of bias. These included protocol registration, multiple database searches, clear entry criteria, inclusion of non-English articles, contacting authors for data, citation searching, and having two reviewers involved in study entry decisions, data extraction and risk of bias assessment.

A significant strength of the findings lies in the diverse range of health settings and countries represented. Although most of the trials were conducted in high-income countries, more than one-third of the trials emerged from upper-middle-income and lower-middle-income nations, highlighting a broad global perspective and the application of the results across varying socioeconomic contexts. All the included trials were real-world evaluations of information interventions. We also employed a novel method in the inclusion of albatross plots, which provide a simple way of displaying data from multiple trials, which is particularly useful when meta-analysis is not feasible.

However, there are some limitations. First, few trials assessed knowledge over the longer term: in certain settings, such as illness prevention or the management of long-term conditions, longer-term increases in knowledge would be a more important indicator of intervention success. Conversely, in other settings, like helping patients to prepare for surgery or a scan, short-term knowledge gains would serve as valid indicators. Secondly, a minority of trials (36%) assessed behaviour outcomes, which in some settings would be the most important indicator of effect. However, in other cases knowledge gain would be both sufficient and the most realistic positive outcome. Thirdly, individual trials were often small and exhibited substantial variation across various study elements. As in our 2022 review, trials were often small (i.e., the median sample size was 120), raising concerns about Type 2 statistical error in trials reporting null effects. Fourthly, a minority of publications included links to the tested animations. Copyright restrictions likely played a role but not being able to view these animations limits the conclusions drawn. For example, it makes it impossible to assess their content, tone or quality, and hinders study replication and the development of effective interventions, which are vital elements of science.

Fifth, only three trials specifically evaluated the effectiveness of animations for people with low health literacy (34, 59, 82), and none looked at effectiveness across different groups in the population, e.g., by education level or income. Notably, none of the trials addressed interventions for individuals with disabilities, such as learning disability, representing a significant gap. Not only could factors such as education, income and disability be mediators of effectiveness, it is also possible that animations could be relatively more effective in less educated groups; this important possibility has not been evaluated.

A final limitation is that the quality of the 88 trials was mixed, with only 16 of them rated as having a low risk of bias.

### 4.3 Implications of the findings

Our findings are consistent with previous systematic reviews (99, 100) which have showed a positive impact of using animations to communicate health information among patients in various healthcare settings. However, our review differs in that we included studies of members of the public and of patients of all age groups, as well as a wider range of intervention settings and outcome categories. Also, the inclusion in the review of studies from UMICs and LMICs highlights the relevance of video animations as informational tools in resource-constrained settings.

### 4.4 Recommendations for future research

There remains a significant need for high-quality randomised controlled trials with transparent reporting, robust randomisation, adequate sample sizes, and the provision of a link to the tested animation. Future research should prioritise the development and evaluation of animation-based interventions tailored to individuals with lower levels of literacy, including minority-language speakers and those with less health literacy. Future research should also consider developing and evaluating animation interventions for these underserved populations to promote inclusivity and health equity. It would also be important for animations in health settings to be developed using guidance on their content, design and delivery, as reported in a recent realist review (101).

It is essential that trials continue to examine how animations are used in real-world healthcare settings and also assess how context affects their impact, especially for explaining complex healthcare procedures. Consequently, research should extend beyond immediate knowledge acquisition to include the assessment of longer-term outcomes. Health behaviours were assessed in a minority of included trials and this aspect is crucial for assessing the potential for animations to have effects beyond increases in knowledge and satisfaction. It would also be helpful for trials in some settings to include a cost-effectiveness evaluation.

Finally, the potential of animation-based interventions is evident, and it is important to continue building a robust evidence base. Practitioners are encouraged to consider animations as part of a broader health education strategy while being aware of the current limitations in research quality and consistency.

## 5 Conclusions

This review covers trials conducted in the OECD countries, upper-middle-income countries (UMICs), and lower-middle-income countries (LMICs). Consistent with our previous review, our analysis reveals predominantly positive impacts on patient knowledge, especially in the short term. Additionally, we observed some beneficial effects on attitudes and cognitions, and the results further indicate positive effects on behaviour. Of the 88 included trials, only two reported statistically significant findings favouring the control group, underscoring the potential of video animations in enhancing patient information delivery.

## Data Availability

The original contributions presented in the study are included in the article/Supplementary Material, further inquiries can be directed to the corresponding author/s.
