# Correction: How effective are video animations as information tools for patients and the general public? An updated systematic review

**DOI:** 10.3389/fdgth.2026.1787856

**Published:** 2026-01-30

**Authors:** Frontiers Production Office

**Affiliations:** Frontiers Media SA, Lausanne, Switzerland

**Keywords:** video animations, information tools, patients, knowledge, attitudes and cognition, behaviours, albatross plot

Supplementary Material Tables 1–3 were omitted. The file(s) have now been published.

The supplementary citations for Supplementary Tables 5–7 have been revised to Supplementary Tables 1–3 throughout the article.

The original version of this article has been updated.

