# Supplementary material for: How effective are video animations as information tools for patients and the general public? An updated systematic review
Source: Front Digit Health. 2026 Jan 2;7:1717044. doi: 10.3389/fdgth.2025.1717044 (PMC12808424; doi:10.3389/fdgth.2025.1717044)
Supplement: Supplementary file 2 [file Table1.docx]

The Effectiveness of Video Animations as Informational Tools

Update of Literature Search Report:

**Information Specialist:**Su Golder PhD, MSc, BSc (Hons), FRSA

**Document Last Updated:** 04 June 2024

Contents

[Information Sources and Methods 2](#_Toc74150680)

[Search Strategies 3](#_Toc74150681)

[Medline (via Ovid) 3](#_Toc74150682)

[Embase (via Ovid) 7](#_Toc74150683)

[PsycINFO (via Ovid) 11](#_Toc74150684)

[Cochrane CENTRAL (via Wiley) 15](#_Toc74150685)

[CINAHL Complete (via EBSCO Host) 17](#_Toc74150686)

[OpenGrey (via http://www.opengrey.eu/) 21](#_Toc74150687)

[Managing Records 22](#_Toc74150688)

[Search Write-Up 23](#_Toc74150689)

# Information Sources and Methods

**Databases and Platforms:**

| **Database:** | **Platform:** |
| --- | --- |
| Medline | (via Ovid) |
| Embase | (via Ovid) |
| PsycINFO | (via Ovid) |
| Cochrane CENTRAL | (via Wiley) |
| CINAHL Complete | (via EBSCO Host) |
| OpenGrey | (via http://www.opengrey.eu/) |

# Search Strategies

## Medline (via Ovid)

| **Database Version & Platform:** | Ovid MEDLINE(R) ALL <1946 to June 03, 2024> |
| --- | --- |
| **Date Range Searched:** | 2021 for date record created |
| **Date of Most Recent Search:** | 04/06/2024 |
| **Records Retrieved:** | 343 |

| **Line Number:** | **Search Strategy:** | **Hits:** |
| --- | --- | --- |
| Ovid MEDLINE(R) ALL <1946 to June 03, 2024> | | |
| 1 | Cartoons as Topic/ | 634 |
| 2 | Caricatures as Topic/ | 283 |
| 3 | Motion Pictures/ | 8561 |
| 4 | ((animation* or computer-animat* or digital-animat* or digitally-animat* or motion comic* or motion-comic* or anime) not "suspended animation").ti,ab,kw,kf. | 4085 |
| 5 | (animated adj6 (film* or video* or visual* or picture* or image* or character*1 or avatar* or cartoon* or webtoon* or web-toon* or web-cartoon* or comic* or web-comic* or webcomic* or caricature* or manga)).ti,ab,kw,kf. | 1026 |
| 6 | ((computer* or digital*) adj4 (animat* or character*1 or avatar* or cartoon* or webtoon* or web-toon* or web-cartoon* or caricature*)).ti,ab,kw,kf. | 975 |
| 7 | (illustrated adj3 (film* or video* or watch*)).ti,ab,kw,kf. | 219 |
| 8 | ((avatar* or cartoon* or webtoon* or web-toon* or web-cartoon* or caricature*) and (film* or watch* or view* or video*)).ti,ab,kw,kf. | 1127 |
| 9 | or/1-8 | 15570 |
| 10 | exp Health/ | 453141 |
| 11 | exp Public Health/ | 9546411 |
| 12 | exp Population Health/ | 41147 |
| 13 | exp Health Education/ | 266282 |
| 14 | exp "Patient Education as Topic"/ | 89065 |
| 15 | exp Consumer Health Information/ | 14283 |
| 16 | Health Communication/ | 3391 |
| 17 | Attitude to Health/ | 85476 |
| 18 | Patient Acceptance of Health Care/ | 56174 |
| 19 | (inform*3 or instruct* or self-instruct* or learn* or e-learn* or self-learn* or educat* or self-educat* or teach* or train* or taught or self-taught or counsel* or advice or advis* or guide* or guidance or understand* or knowledge* or self-knowledge).ti,ab,kw,kf. | 5430798 |
| 20 | or/10-19 | 13261364 |
| 21 | 9 and 20 | 7117 |
| 22 | Randomized Controlled Trials as Topic/ | 170582 |
| 23 | Randomized Controlled Trial/ | 614384 |
| 24 | Random Allocation/ | 107249 |
| 25 | Double-Blind Method/ | 178784 |
| 26 | Single-Blind Method/ | 33548 |
| 27 | Clinical Trial/ | 540010 |
| 28 | clinical trial, phase i.pt. | 26020 |
| 29 | clinical trial, phase ii.pt. | 41363 |
| 30 | clinical trial, phase iii.pt. | 22789 |
| 31 | clinical trial, phase iv.pt. | 2505 |
| 32 | controlled clinical trial.pt. | 95542 |
| 33 | randomized controlled trial.pt. | 614384 |
| 34 | multicenter study.pt. | 347592 |
| 35 | clinical trial.pt. | 540010 |
| 36 | exp "Clinical Trials as Topic"/ | 392910 |
| 37 | or/22-36 | 1610229 |
| 38 | (clinical adj trial$).tw. | 514090 |
| 39 | ((singl$ or doubl$ or treb$ or tripl$) adj (blind$3 or mask$3)).tw. | 205496 |
| 40 | Placebos/ | 35964 |
| 41 | placebo$.tw. | 256841 |
| 42 | randomly allocated.tw. | 38744 |
| 43 | (allocated adj2 random$).tw. | 42671 |
| 44 | or/38-43 | 829146 |
| 45 | Research Design/ | 128215 |
| 46 | (experiment* adj5 (research* or design* or group* or psychology)).tw,kw. | 237881 |
| 47 | (experiment*1 adj3 control*1 adj8 (group* or divide* or assign* or allocate* or random*)).tw,kw. | 1923 |
| 48 | (control* adj3 condition*).tw,kw. | 64753 |
| 49 | (research adj4 design*).tw,kw. | 63844 |
| 50 | (DoE adj6 (experiment* or approach*)).tw,kw. | 1822 |
| 51 | (counterbalanc* or counter-balanc* or "counter balanc*").tw,kw. | 15334 |
| 52 | (latin square* or latin-square*).tw,kw. | 5975 |
| 53 | ((independent or repeated) adj3 measure*).tw,kw. | 78438 |
| 54 | (independent-measure* or repeated-measure*).tw,kw. | 63717 |
| 55 | (between-subject* or "between subject*" or within-subject* or "within subject*" or between-group* or "between group*").tw,kw. | 212672 |
| 56 | (matched pair* or matched-pair*).tw,kw. | 12349 |
| 57 | (mixed adj4 (research* or method*)).tw,kw. | 55697 |
| 58 | (multiple adj4 method*).tw,kw. | 31806 |
| 59 | (mixed-method* or multiple-method* or multi-method* or multimethod*).tw,kw. | 58245 |
| 60 | ((closed ended or closed-ended) and (open ended or open-ended)).tw,kw. | 746 |
| 61 | (qualitative* and quantitative*).tw,kw. | 133571 |
| 62 | or/45-61 | 975162 |
| 63 | 21 and (37 or 44 or 62) | 1171 |
| 64 | letter/ | 1256252 |
| 65 | editorial/ | 693488 |
| 66 | news/ | 225511 |
| 67 | exp historical article/ | 411319 |
| 68 | comment/ | 1036442 |
| 69 | (letter or comment*).ti. | 200346 |
| 70 | (comment or conference or letter).pt. | 1770041 |
| 71 | or/64-70 | 2919160 |
| 72 | 63 not 71 | 1152 |
| 73 | (2021* or 2022* or 2023* or 2024*).dt. | 5410576 |
| 74 | 72 and 73 | 343 |

| **Key to Search Symbols / Operators:** | / or .sh. = indexing term: Medical Subject Heading (MeSH)  exp = exploded indexing term  * or $ = truncation  *n = truncated by number of characters specified  ? = replaces zero or one character  ti,ab,kw,kf = searches in title, abstract, keyword, or keyfield fields  pt,tw = publication type, textword fields  adj3 = terms within three words of each other (any order) |
| --- | --- |
| **Limits and Restrictions:** | Papers limited by study type to either randomised or clinical trials, or mixed methods / experimental studies.  Irrelevant paper types removed from this database: letters, editorials, comments, conference papers, news, or historical articles. |
| **Search Filters:** | A study design search filter developed by SIGN which was designed to restrict retrieval to randomised controlled trials and clinical trials was included in the strategy. |

## Embase (via Ovid)

| **Database Version & Platform:** | Embase <1974 to 2024 June 03> |
| --- | --- |
| **Date Range Searched:** | 2021 for date record created |
| **Date of Most Recent Search:** | 04/06/2024 |
| **Records Retrieved:** | 436 |

| **Line Number:** | **Search Strategy:** | **Hits:** |
| --- | --- | --- |
| Embase <1974 to 2024 June 03> |  |  |
| 1 | (art/ or computer graphics/ or medical illustration/) and (movie/ or television/ or videorecording/) | 990 |
| 2 | ((animation* or computer-animat* or digital-animat* or digitally-animat* or motion comic* or motion-comic* or anime) not "suspended animation").ti,ab,kw. | 5396 |
| 3 | (animated adj6 (film* or video* or visual* or picture* or image* or character*1 or avatar* or cartoon* or webtoon* or web-toon* or web-cartoon* or comic* or web-comic* or webcomic* or caricature* or manga)).ti,ab,kw. | 1351 |
| 4 | ((computer* or digital*) adj4 (animat* or character*1 or avatar* or cartoon* or webtoon* or web-toon* or web-cartoon* or caricature*)).ti,ab,kw. | 1163 |
| 5 | (illustrated adj3 (film* or video* or watch*)).ti,ab,kw. | 368 |
| 6 | ((avatar* or cartoon* or webtoon* or web-toon* or web-cartoon* or caricature*) and (film* or watch* or view* or video*)).ti,ab,kw. | 1437 |
| 7 | or/1-6 | 9191 |
| 8 | exp health/ | 945369 |
| 9 | exp public health/ | 243392 |
| 10 | exp population health/ | 6875 |
| 11 | exp health education/ | 393901 |
| 12 | exp patient education/ | 128933 |
| 13 | exp consumer health information/ | 4340 |
| 14 | attitude to health/ | 134671 |
| 15 | patient attitude/ | 79549 |
| 16 | (health* adj3 (educat* or public* or population* or communit* or knowledge* or literate or literacy or attitude*)).ti,ab,kw. | 724018 |
| 17 | (inform*3 or instruct* or self-instruct* or learn* or e-learn* or self-learn* or educat* or self-educat* or teach* or train* or taught or self-taught or counsel* or advice or advis* or guide* or guidance or understand* or knowledge* or self-knowledge).ti,ab,kw. | 7054112 |
| 18 | or/8-17 | 8219616 |
| 19 | 7 and 18 | 5106 |
| 20 | clinical trial/ | 1083389 |
| 21 | randomized controlled trial/ | 824709 |
| 22 | controlled clinical trial/ | 473279 |
| 23 | multicenter study/ | 393919 |
| 24 | phase 3 clinical trial/ | 75948 |
| 25 | phase 4 clinical trial/ | 7353 |
| 26 | exp randomization/ | 99733 |
| 27 | single blind procedure/ | 54982 |
| 28 | double blind procedure/ | 219670 |
| 29 | crossover procedure/ | 78225 |
| 30 | placebo/ | 413525 |
| 31 | randomi?ed controlled trial$.tw. | 347548 |
| 32 | rct.tw. | 57908 |
| 33 | (random$ adj2 allocat$).tw. | 57545 |
| 34 | single blind$.tw. | 33228 |
| 35 | double blind$.tw. | 252507 |
| 36 | ((treble or triple) adj blind$).tw. | 2124 |
| 37 | placebo$.tw. | 380120 |
| 38 | prospective study/ | 921117 |
| 39 | or/20-38 | 3062517 |
| 40 | methodology/ | 1642941 |
| 41 | experimental design/ | 26975 |
| 42 | latin square design/ | 448 |
| 43 | qualitative research/ and (quantitative study/ or quantitative analysis/) | 6257 |
| 44 | (experiment* adj5 (research* or design* or group* or psychology)).tw,kw. | 292631 |
| 45 | (control* adj3 condition*).tw,kw. | 76310 |
| 46 | (research adj4 design*).tw,kw. | 76159 |
| 47 | (DoE adj6 (experiment* or approach*)).tw,kw. | 2241 |
| 48 | (counterbalanc* or counter-balanc* or "counter balanc*").tw,kw. | 19264 |
| 49 | (latin square* or latin-square*).tw,kw. | 6374 |
| 50 | ((independent or repeated) adj3 measure*).tw,kw. | 110524 |
| 51 | (independent-measure* or repeated-measure*).tw,kw. | 90782 |
| 52 | (between-subject* or "between subject*" or within-subject* or "within subject*" or between-group* or "between group*").tw,kw. | 340399 |
| 53 | (matched pair* or matched-pair*).tw,kw. | 18573 |
| 54 | (mixed adj4 (research* or method*)).tw,kw. | 65594 |
| 55 | (multiple adj4 method*).tw,kw. | 51654 |
| 56 | (mixed-method* or multiple-method* or multi-method* or multimethod*).tw,kw. | 66124 |
| 57 | ((closed ended or closed-ended) and (open ended or open-ended)).tw,kw. | 921 |
| 58 | (qualitative* and quantitative*).tw,kw. | 167832 |
| 59 | or/40-58 | 2765554 |
| 60 | 19 and (39 or 59) | 1275 |
| 61 | letter/ or case report/ or case study/ | 4086314 |
| 62 | (letter or comment*).ti. | 245652 |
| 63 | (comment or letter or editorial or note).pt. | 3119310 |
| 64 | or/61-63 | 5937050 |
| 65 | 60 not 64 | 1248 |
| 66 | remove duplicates from 65 | 1237 |
| 67 | (2021* or 2022* or 2023* or 2024*).dc. | 7154814 |
| 68 | 66 and 67 | 436 |

| **Key to Search Symbols / Operators:** | / or .sh. = indexing term: Emtree headings.  exp = exploded indexing term  * or $ = truncation  *n = truncated by number of characters specified  ? = replaces zero or one character  ti,ab,kw = searches in title, abstract, keyword, or fields  pt,tw = publication type, textword fields  adj3 = terms within three words of each other (any order) |
| --- | --- |
| **Limits and Restrictions:** | Papers limited by study type to either randomised or clinical trials, or mixed methods / experimental studies.  Irrelevant paper types removed from this database: letters, case reports, case studies, comments, editorials, and notes.  Embase records were de-duplicated against each other (using Ovid’s inbuilt de-duplication feature) before the records were downloaded. |
| **Search Filters:** | A study design search filter developed by SIGN which was designed to restrict retrieval to randomised controlled trials and clinical trials was included in the strategy. |

## PsycINFO (via Ovid)

| **Database Version & Platform:** | APA PsycInfo <1806 to May Week 5 2024> |
| --- | --- |
| **Date Range Searched:** | 2021 for date record created |
| **Date of Most Recent Search:** | 04/06/2024 |
| **Records Retrieved:** | 156 |

| **Line Number:** | **Search Strategy:** | **Hits:** |
| --- | --- | --- |
| APA PsycInfo <1806 to May Week 5 2024> | |  |
| 1 | exp Animation/ | 887 |
| 2 | "Cartoons (Humor)"/ | 682 |
| 3 | Cartoons as Topic.mh. | 160 |
| 4 | Caricatures as Topic.mh. | 61 |
| 5 | ((animation* or computer-animat* or digital-animat* or digitally-animat* or motion comic* or motion-comic* or anime) not "suspended animation").ti,ab,id. | 3037 |
| 6 | (animated adj6 (film* or video* or visual* or picture* or image* or character*1 or avatar* or cartoon* or webtoon* or web-toon* or web-cartoon* or comic* or web-comic* or webcomic* or caricature* or manga)).ti,ab,id. | 968 |
| 7 | ((computer* or digital*) adj4 (animat* or character*1 or avatar* or cartoon* or webtoon* or web-toon* or web-cartoon* or caricature*)).ti,ab,id. | 1000 |
| 8 | (illustrated adj3 (film* or video* or watch*)).ti,ab,id. | 69 |
| 9 | ((avatar* or cartoon* or webtoon* or web-toon* or web-cartoon* or caricature*) and (film* or watch* or view* or video*)).ti,ab,id. | 1615 |
| 10 | or/1-9 | 6390 |
| 11 | exp Health/ or Health.mh. | 487859 |
| 12 | exp Public Health/ or Public Health.mh. | 41966 |
| 13 | Population Health.mh. | 86 |
| 14 | exp Health Education/ or Health Education.mh. | 28734 |
| 15 | Patient Education as Topic.mh. | 11214 |
| 16 | Consumer Health Information.mh. | 930 |
| 17 | Health Communication.mh. | 876 |
| 18 | Attitude to Health.mh. | 25524 |
| 19 | Patient Acceptance of Health Care.mh. | 12640 |
| 20 | (health* adj3 (educat* or public* or population* or communit* or knowledge* or literate or literacy or attitude*)).ti,ab,id. | 163554 |
| 21 | (inform*3 or instruct* or self-instruct* or learn* or e-learn* or self-learn* or educat* or self-educat* or teach* or train* or taught or self-taught or counsel* or advice or advis* or guide* or guidance or understand* or knowledge* or self-knowledge).ti,ab,id. | 2331527 |
| 22 | or/11-21 | 2647330 |
| 23 | 10 and 22 | 3581 |
| 24 | limit 23 to "0300 clinical trial" | 30 |
| 25 | Randomized Controlled Trials as Topic.mh. | 10337 |
| 26 | Randomized Controlled Trial/ or Randomized Controlled Trial.mh. | 1075 |
| 27 | Random Allocation.mh. | 8276 |
| 28 | Double-Blind Method.mh. | 19743 |
| 29 | Single-Blind Method.mh. | 3214 |
| 30 | Clinical Trials/ or Clinical Trial.mh. | 12359 |
| 31 | controlled clinical trial.ab,id. | 1465 |
| 32 | randomi#ed controlled trial.mp. or RCT.ab,id. | 35246 |
| 33 | multicenter study.ab,id. | 1488 |
| 34 | clinical trial.ab,id. | 17287 |
| 35 | Clinical Trials as Topic.mh. | 9808 |
| 36 | or/25-35 | 98608 |
| 37 | (clinical adj trial$).tw. | 44773 |
| 38 | ((singl$ or doubl$ or treb$ or tripl$) adj (blind$3 or mask$3)).tw. | 29927 |
| 39 | Placebo/ or Placebos.mh. | 10926 |
| 40 | placebo$.tw. | 45532 |
| 41 | randomly allocated.tw. | 4606 |
| 42 | (allocated adj2 random$).tw. | 4870 |
| 43 | or/37-42 | 96254 |
| 44 | exp experimental design/ | 64395 |
| 45 | Research Design.mh. | 14815 |
| 46 | (experiment* adj5 (research* or design* or group* or psychology)).ti,ab,id. | 94617 |
| 47 | (experiment*1 adj3 control*1 adj8 (group* or divide* or assign* or allocate* or random*)).ti,ab,id. | 591 |
| 48 | (control* adj3 condition*).ti,ab,id. | 30455 |
| 49 | (research adj4 design*).ti,ab,id. | 48991 |
| 50 | (DoE adj6 (experiment* or approach*)).ti,ab,id. | 37 |
| 51 | (counterbalanc* or counter-balanc* or "counter balanc*").ti,ab,id. | 6985 |
| 52 | (latin square* or latin-square*).ti,ab,id. | 590 |
| 53 | ((independent or repeated) adj3 measure*).ti,ab,id. | 25525 |
| 54 | (independent-measure* or repeated-measure*).ti,ab,id. | 22512 |
| 55 | (between-subject* or "between subject*" or within-subject* or "within subject*" or between-group* or "between group*").ti,ab,id. | 61204 |
| 56 | (matched pair* or matched-pair*).ti,ab,id. | 1959 |
| 57 | (mixed adj4 (research* or method*)).ti,ab,id. | 43717 |
| 58 | (multiple adj4 method*).ti,ab,id. | 9426 |
| 59 | (mixed-method* or multiple-method* or multi-method* or multimethod*).ti,ab,id. | 49549 |
| 60 | ((closed ended or closed-ended) and (open ended or open-ended)).ti,ab,id. | 504 |
| 61 | (qualitative* and quantitative*).ti,ab,id. | 55736 |
| 62 | or/44-61 | 402403 |
| 63 | 23 and (24 or 36 or 43 or 62) | 633 |
| 64 | remove duplicates from 63 | 631 |
| 65 | (2021* or 2022* or 2023* or 2024*).up. | 657964 |
| 66 | 64 and 65 | 156 |

| **Key to Search Symbols / Operators:** | / or .sh. = indexing term: Thesaurus of Psychological Index Terms  exp = exploded indexing term  .mh. = indexing term: Medical Subject Heading (MeSH)  * or $ = truncation  *n = truncated by number of characters specified  ? = replaces zero or one character  ti,ab,id,hw = searches in title, abstract, key concepts, or heading word fields  adj3 = terms within three words of each other (any order) |
| --- | --- |
| **Limits and Restrictions:** | Papers limited by study type to either randomised or clinical trials, or mixed methods / experimental studies.  PsycINFO records were de-duplicated against each other (using Ovid’s inbuilt de-duplication feature) before the records were downloaded. |

## Cochrane CENTRAL (via Wiley)

| **Database Version & Platform:** | Cochrane CENTRAL (via Wiley) |
| --- | --- |
| **Date Range Searched:** | 2021 |
| **Date of Most Recent Search:** | 06/06/2024 |
| **Records Retrieved:** | 525 |

| **Line Number:** | **Search Strategy:** | **Hits:** |
| --- | --- | --- |
| #1 | [mh ^"Cartoons as Topic"] | 49 |
| #2 | [mh ^"Caricatures as Topic"] | 0 |
| #3 | [mh ^"Motion Pictures"] | 222 |
| #4 | ((animation* OR computer NEXT animat* OR digital NEXT animat* OR digitally NEXT animat* OR motion comic? OR motion NEXT comic? OR anime) NOT "suspended animation"):ti,ab,kw | 643 |
| #5 | (animated NEAR/6 (film* OR video* OR visual* OR picture* OR image* OR character? OR avatar* OR cartoon* OR webtoon? OR web NEXT toon? OR web NEXT cartoon? OR comic* OR web NEXT comic* OR webcomic* OR caricature* OR manga)):ti,ab,kw | 356 |
| #6 | ((computer* OR digital*) NEAR/4 (animat* OR character? OR avatar* OR cartoon* OR webtoon? OR web NEXT toon? OR web NEXT cartoon? OR caricature*)):ti,ab,kw | 159 |
| #7 | (illustrated NEAR/3 (film* OR video* OR watch*)):ti,ab,kw | 18 |
| #8 | ((avatar* OR cartoon* OR webtoon* OR web NEXT toon* OR web NEXT cartoon* OR caricature*) and (film* OR watch* OR view* OR video*)):ti,ab,kw | 404 |
| #9 | {OR #1-#8} | 1598 |
| #10 | [mh Health] | 14712 |
| #11 | [mh "Public Health"] | 636863 |
| #12 | [mh "Population Health"] | 1064 |
| #13 | [mh "Health Education"] | 26028 |
| #14 | [mh "Patient Education as Topic"] | 10867 |
| #15 | [mh "Consumer Health Information"] | 899 |
| #16 | [mh ^"Health Communication"] | 380 |
| #17 | [mh ^"Attitude to Health"] | 3463 |
| #18 | [mh ^"Patient Acceptance of Health Care"] | 4271 |
| #19 | (health* NEAR/3 (educat* OR public* OR population* OR communit* OR knowledge* OR literate OR literacy OR attitude*)):ti,ab,kw | 67908 |
| #20 | (inform? OR informed OR instruct? OR instructed OR instructional OR self NEXT instruct* OR learn* OR e NEXT learn* OR self NEXT learn* OR educat* OR self NEXT educat* OR teach* OR train* OR taught OR self NEXT taught OR counsel* OR advice OR advis* OR guide* OR guidance OR understand* OR knowledge* OR self NEXT knowledge):ti,ab,kw | 540658 |
| #21 | {OR #10-#20} | 1049504 |
| #22 | #9 AND #21 in Trials | **525** |

| **Key to Search Symbols / Operators:** | mh = exploded indexing term: Medical Subject Heading (MeSH)  mh ^ = unexploded indexing term  * truncation  ? = one additional character  :ti,ab,kw = searches in title, abstract, or keyword fields  NEAR/3 = terms within three words of each other (any order)  NEXT = words must be next to each other |
| --- | --- |

## CINAHL Complete (via EBSCO Host)

| **Database Version & Platform:** | CINAHL Complete (via EBSCO Host) |
| --- | --- |
| **Date Range Searched:** | 2021 for entry date of record |
| **Date of Most Recent Search:** | 05/06/2024 |
| **Records Retrieved:** | 306 |

| **Line Number:** | **Search Strategy:** | **Hits:** |
| --- | --- | --- |
| S58 | S56 AND S57 | **306** |
| S57 | EM 2021- | 1,050,322 |
| S56 | S21 AND S55 | 1,094 |
| S55 | S36 OR S54 | 2,298,535 |
| S54 | S37 OR S38 OR S39 OR S40 OR S41 OR S42 OR S43 OR S44 OR S45 OR S46 or S47 OR S48 OR S49 OR S50 OR S51 OR S52 OR S53 | 253,455 |
| S53 | TI ( qualitative* and quantitative* ) OR AB ( qualitative* and quantitative* ) | 36,534 |
| S52 | TI ( (closed ended or closed-ended) and (open ended or open-ended) ) OR AB ( (closed ended or closed-ended) and (open ended or open-ended) ) | 1,043 |
| S51 | TI ( mixed-method* or multiple-method* or multi-method* or multimethod* ) OR AB ( mixed-method* or multiple-method* or multi-method* or multimethod* ) | 34,357 |
| S50 | TI multiple N4 method* OR AB multiple N4 method* | 9,066 |
| S49 | TI ( mixed N4 (research* or method*) ) OR AB ( mixed N4 (research* or method*) ) | 33,018 |
| S48 | TI ( matched pair* or matched-pair* ) OR AB ( matched pair* or matched-pair* ) | 5,033 |
| S47 | TI ( between-subject* or "between subject*" or within-subject* or "within subject*" or between-group* or "between group*" ) OR AB ( between-subject* or "between subject*" or within-subject* or "within subject*" or between-group* or "between group*" ) | 66,235 |
| S46 | TI ( independent-measure* or repeated-measure* ) OR AB ( independent-measure* or repeated-measure* ) | 26,090 |
| S45 | TI ( (independent or repeated) N3 measure* ) OR AB ( (independent or repeated) N3 measure* ) | 30,863 |
| S44 | TI ( latin square* or latin-square* ) OR AB ( latin square* or latin-square* ) | 278 |
| S43 | TI ( counterbalanc* or counter-balanc* or "counter balanc*" ) OR AB ( counterbalanc* or counter-balanc* or "counter balanc*" ) | 3,570 |
| S42 | TI ( DoE N6 (experiment* or approach*) ) OR AB ( DoE N6 (experiment* or approach*) ) | 1,820 |
| S41 | TI research N4 design* OR AB research N4 design* | 33,146 |
| S40 | TI control* N3 condition* OR AB control* N3 condition* | 13,597 |
| S39 | TI ( experiment# N3 control# N8 (group* or divide* or assign* or allocate* or random*) ) OR AB ( experiment# N3 control# N8 (group* or divide* or assign* or allocate* or random*) ) | 453 |
| S38 | TI ( experiment* N5 (research* or design* or group* or psychology) ) OR AB ( experiment* N5 (research* or design* or group* or psychology) ) | 47,113 |
| S37 | MH Research Design | 0 |
| S36 | S22 OR S23 OR S24 OR S25 OR S26 OR S27 OR S28 OR S29 OR S30 OR S32 OR S33 OR S34 OR S35 | 2,155,564 |
| S35 | TX allocat* random* | 38,994 |
| S34 | MH "Quantitative Studies" | 39,990 |
| S33 | MH "Placebos" | 14,465 |
| S32 | TX placebo* | 186,051 |
| S31 | TX random* allocat* | 38,994 |
| S30 | MH "Random Assignment" | 85,687 |
| S29 | TX randomi* control* trial* | 480,091 |
| S28 | TX (singl* N1 blind*) or (singl* N1 mask*) | 33,678 |
| S27 | TX (doubl* N1 blind*) or (doubl* N1 mask*) | 1,465,587 |
| S26 | TX (tripl* N1 blind*) or (tripl* N1 mask*) | 1,960 |
| S25 | TX (trebl* N1 blind*) or (trebl* N1 mask*) | 24 |
| S24 | TX clinic* N1 trial* | 555,441 |
| S23 | PT clinical trial | 114,374 |
| S22 | MH "Clinical Trials+" | 357,421 |
| S21 | S9 AND S20 | 3,348 |
| S20 | S10 OR S11 OR S12 OR S13 OR S14 OR S15 OR S16 OR S17 OR S18 OR S19 | (3,265,101 |
| S19 | TI ( inform* or informed or informing or instruct* or self-instruct* or learn* or e-learn* or self-learn* or educat* or self-educat* or teach* or train* or taught or self-taught or counsel* or advice or advis* or guide* or guidance or understand* or knowledge* or self-knowledge ) OR AB ( inform* or informed or informing or instruct* or self-instruct* or learn* or e-learn* or self-learn* or educat* or self-educat* or teach* or train* or taught or self-taught or counsel* or advice or advis* or guide* or guidance or understand* or knowledge* or self-knowledge ) | 1,952,714 |
| S18 | TI ( health* N3 (educat* or public* or population* or communit* or knowledge* or literate or literacy or attitude*) ) OR AB ( health* N3 (educat* or public* or population* or communit* or knowledge* or literate or literacy or attitude*) ) | 276,053 |
| S17 | MH Patient Acceptance of Health Care | 0 |
| S16 | MH Health Communication | 0 |
| S15 | MH "Consumer Health Information+" | 21,570 |
| S14 | MH "Patient Education as Topic+" | 0 |
| S13 | MH "Health Education+" | 214,532 |
| S12 | MH "Population Health+" | 2,444 |
| S11 | MH "Public Health+" | 1,469,583 |
| S10 | MH "Health+" | 475,098 |
| S9 | S1 OR S2 OR S3 OR S4 OR S5 OR S6 OR S7 OR S8 | 8,755 |
| S8 | TI ( (avatar* or cartoon* or webtoon* or web-toon* or web-cartoon* or caricature*) and (film* or watch* or view* or video*) ) OR AB ( (avatar* or cartoon* or webtoon* or web-toon* or web-cartoon* or caricature*) and (film* or watch* or view* or video*) ) | 340 |
| S7 | TI ( illustrated N3 (film* or video* or watch*) ) OR AB ( illustrated N3 (film* or video* or watch*) ) | 42 |
| S6 | TI ( (computer* or digital*) N4 (animat* or character# or avatar* or cartoon* or webtoon* or web-toon* or web-cartoon* or caricature*) ) OR AB ( (computer* or digital*) N4 (animat* or character# or avatar* or cartoon* or webtoon* or web-toon* or web-cartoon* or caricature*) ) | 270 |
| S5 | TI ( animated N6 (film* or video* or visual* or picture* or image* or character# or avatar* or cartoon* or webtoon* or web-toon* or web-cartoon* or comic* or web-comic* or webcomic* or caricature* or manga) ) OR AB ( animated N6 (film* or video* or visual* or picture* or image* or character# or avatar* or cartoon* or webtoon* or web-toon* or web-cartoon* or comic* or web-comic* or webcomic* or caricature* or manga) ) | 383 |
| S4 | TI ( animation* or computer-animat* or digital-animat* or digitally-animat* or motion comic* or motion-comic* or anime) OR AB ( animation* or computer-animat* or digital-animat* or digitally-animat* or motion comic or motion-comic or anime) NOT TX "suspended animation" | 1,029 |
| S3 | MH Motion Pictures | 7,195 |
| S2 | MH "Caricatures as Topic" | 0 |
| S1 | MH "Cartoons as Topic" | 0 |

| **Key to Search Symbols / Operators:** | MH = indexing term: exact Medical Subject Heading (MeSH)  where + is used at the end of a MeSH term, this explodes the term  * = truncation  "" = exact phrase search  ? = replaces one character within a word  # = optional character within a word  TI = searches in title field  AB = searches in title field  TX = search across all text  N3 = terms within three words of each other (any order) |
| --- | --- |
| **Limits and Restrictions:** | Papers limited by study type to either randomised or clinical trials, or mixed methods / experimental studies. |
| **Search Filters:** | A study design search filter developed by Mark Clowes which was designed to restrict retrieval to randomised controlled trials and clinical trials was included in the strategy. |

## OpenGrey (via http://www.opengrey.eu/)

| **Database Version & Platform:** | OpenGrey (via http://www.opengrey.eu/) |
| --- | --- |
| **Date Range Searched:** | No limits by date. |
| **Date of Most Recent Search:** | DD/MM/YYYY |
| **Records Retrieved:** | n results of which n were relevant |

| **Search Strategy:** | **Hits:** |
| --- | --- |
| ((animat* NEAR/8 (computer* OR digital* OR film* OR video* OR visual* OR picture* OR image* OR character* OR avatar* OR cartoon* OR comic* OR webcomic* OR webtoon* OR caricature* OR manga)) NOT "suspended animation") | **[n of which n were relevant]** |

| **Key to Search Symbols / Operators:** | * = truncation  “” = exact phrase  NEAR/n = within a specified number of words |
| --- | --- |

# Managing Records

| **Databases Searched:** | **Date of Search:** | **Number of Results:** |
| --- | --- | --- |
| Medline (via Ovid) | 04/06/2024 | 343 |
| Embase (via Ovid) | 04/06/2024 | 436 |
| PsycINFO (via Ovid) | 04/06/2024 | 156 |
| Cochrane CENTRAL (via Wiley) | 05/06/2024 | 525 |
| CINAHL Complete (via EBSCO Host) | 05/06/2024 | 306 |
| OpenGrey (via http://www.opengrey.eu/) | DD/MM/YYYY | n |
| **Total results after deduplication** | **1220 (before deduplication 1766)** | |

| **Deduplication Process:** | On Embase and PsycINFO, Ovid’s inbuilt de-duplication features were used. This means that only unique citations were downloaded from each of these databases.  EndNote’s default settings for de-duplication was used to de-duplicate the records – records marked as duplicates were checked by eye. Various iterations of EndNote fields were compared against each other in a further manual process of de-duplication, with records marked as duplicates checked by eye. |
| --- | --- |
