# Supplementary material for: How effective are video animations as information tools for patients and the general public? An updated systematic review
Source: Front Digit Health. 2026 Jan 2;7:1717044. doi: 10.3389/fdgth.2025.1717044 (PMC12808424; doi:10.3389/fdgth.2025.1717044)
Supplement: Supplementary file 3 [file Table2.docx]

### Table 5: Category 1 - Explaining medical or surgical procedures (40 trials)

| **Author, year, country** | **Study design** | **Participants, Setting, Education level between the 2 groups** | **Age, Mean (SD) / %Male** | **Total sample;**  **Intervention descriptor & sample size (I);**  **Control descriptor & sample size (C)** | **Intervention details and link (if provided)** | **Results (Intervention vs Control)** (**Knowledge = knowledge or understanding. Attitudes & Cognitions = satisfaction or self-efficacy or confidence in decision, etc. Behaviours = behaviours or skills or intended behaviours), p value**  | | | **Group favoured** |
| --- | --- | --- | --- | --- | --- | --- | --- | --- | --- |
|  |  |  |  |  |  | **Knowledge** | **Attitudes & Cognitions** | **Behaviours** |  |
| Bowers, 2017, Canada(72) | RCT | Adults undergoing first-time peripherally  Inserted central venous catheter (PICC), Hickman catheter, peripheral angioplasty with or without stenting, or  Endovascular aneurysm repair.  Education level NR | Age: 60.9  Male: 62% | N= 93  I= 49  Multimedia film + Usual care (verbal consent information given by a medical student)  C= 44  Usual care | Multimedia film loaded onto a tablet computer lasting 2 minutes, featuring different films for each procedure.  Link NR. | Total comprehension (during the consent process):  Means not stated.  I n=49; C n = 44.  ANOVA I > C (F = 9.14; p=0.003) on comprehension. | Satisfaction (during the consent process):  Means not stated.  I n=49; C n = 44.  ANOVA I > C (F = 44.1;  p<0.0001). | Not assessed. | **Knowledge:** Favours animation**.**  **Attitudes and Cognitions:** Favours animation**.** |
| Bozkul, 2024, Turkey(77) | RCT (3 arms) | Children (aged 7-12) undergoing planned surgery.  Education level NR | Age:  I = 7.3 (0.5); C1 = 7.6 (2.3);  C2 = 7.6 (1.2)  Male:  I = 93%;  C1 = 77%; C2 = 73% | N= 90  I= 30  Animation +  Usual care (‘routine training in the clinic’)  C1= 30  Short film + Usual care  C2= 30  Usual care | Animation + video on preparation for surgery + ‘routine training in the clinic’ (ie tour of clinic);  length not reported.  Viewed once only.  Link NR. | Not assessed. | Child’s satisfaction scale (0-10) (postoperative period), mean(SD)    I = 8.3 (0.16) (n=30);  C1 = 9.3 (0.18) (n=30);  C2 = 4.7 (0.32) (n=30).  I < C1, p<0.0001.  I > C2, p<0.0001. | Not assessed. | **Attitudes & Cognitions:** Animation better than Control. Short film better than animation**.** |
| Can, 2024, Turkey (94) | RCT | Patients who had undergone ureteroscopic lithotripsy  No statistically significant difference in education level | Age:  I = 45.8 (13.3)  C = 43.5 (12.2)  Male: 61% | N=156  I= 74  Video animated  Information + verbal and written information  C= 82  Verbal and written information | Patients viewed the video on a computer located in a side room adjacent to the intervention room, with the assistance of a single physician  Link  https://patients.uroweb.org/videos/double-j-stent-video/ | Not assessed. | Satisfaction with the procedure (5-point  Likert scale) mean (SD):  Post- intervention:  I = 4.1 (0.9) (n=74);  C = 2.6 (1.0) (n=82);  p<0.001.  Tolerability (how tolerable was the procedure?) (5-point Likert scale  Mean (SD): Post-intervention  I: 3.7 (0.9) (n=74);  C: 2.7 (1.0) (n=82);  p<.001. | Not assessed. | **Attitudes & Cognitions:** Favours animation on both measures. |
| Chanthawong, 2021, Thailand(65) | RCT (3 arms) | Adults due to have planned surgery.  No statistically significant difference in education level | Age:  I = 50.6;  C1 = 45.8; C2 = 45.88  Male: I = 32.9%  C1 = 31.5%  C2 = 42.1% | N=249  I = 83  Animation + Usual care (Verbal anaesthetic information)  C1 = 83  Brochure + Usual care  C2 = 83  Usual care | In-person anaesthetic information + animation lasting 5 minutes, provided on a tablet.  Viewed once only.  Link NR. | Knowledge scores out of 24 points: mean (SD).  For all knowledge outcomes: I n=76; C1 n=73; C2 n=76.  Pre-visit:  I = 16.3 (3.5); C1 = 16.9 (3.5); C2 = 15.7 (3.8).  Immediately after visit:  I = 20.5 (3.3); C1 = 21.1 (3.3); C2 = 18.4 (3.8).  I vs C1, MD 0.54 (95%CI –0.83 to 1.92);  p= NS.  I vs C2, MD 2.12 (95% CI 0.76 to 3.48);  p<0.001.    Day of surgery:  I = 20.2 (3.4); C1 = 20.7 (3.4); C2 = 18.7 (3.3).  I vs C1, MD 0.71 (95% CI –0.59 to 2.01);  p=NS.  I vs C2, MD 1.36 (95% CI 0.07 to 2.64);  p=0.035. | Satisfaction with pre-anaesthetic information (scale of 1 to 10) (recorded 1 day after visit): median (IQR)  I = 9.6 (1.4) (n=76);  C1 = 9.5 (1.0) (n=73); C2 = 9.0 (1.7) (n=76), p=NS. | Not assessed. | **Knowledge:** Favours animation. compared to standard information, but no different to brochure group.  **Attitudes & Cognitions:** No difference between animation and brochure or standard information. |
| Cornoiu, 2011, Australia(74) | RCT (3 Arms) | Adults due to have planned knee arthroscopy.  No statistically significant difference in education level | Age:  I= 45 (SD 17); C1 = 44 (11); C2 = 41 (14)  Males %:  I: 45%; C1:67%’ C2: 81% | N= 61  I= 22 Multimedia, including 3D animation    C1= 18  Verbal consent information (scripted)  C2= 21  Pamphlet (1 page on information using plain English, grade 8 or below). All text and no images | Multimedia education module for knee arthroscopy, included a mixture of voice, text, photographs, and 3D computer animation. 3D animations covered all aspects of core information. The text included in the module was identical to the script developed for the  verbal consent arm.  Link NR. | For all knowledge outcomes: I n=22; C1 n=18; C2 n=21.  Information recall, 10 questions (maximum 100%): mean (SD)  Stage 1 post consent (3-6 weeks pre-surgery:  I: 98% (5%);  C1: 88% (14%);  C2: 76% (28%);  (p<0.05).  Stage 2 (day of surgery):  I: 92% (SD not reported); C1: 77% (SD not reported); C2: 72% (SD not reported).  (p<0.05).  Stage 3 (3-6 weeks post-surgery):  I: 96% (SD not reported);  C1: 86% (SD not reported);  C2: 74% (SD not reported).  (p<0.05).  NB: not reported SDs could be read off the bar chart if needed.  At all 3 stages, knowledge scores in I group were higher than scores in C1, and higher than scores in C2 (p<0.05 but other statistics not reported). | For satisfaction outcomes: I n=22; C1 n=18; C2 n=21.  Satisfaction with information (4 point scale, each with 5 Likert response options),  (timing not reported) mean (SD):  I: 6.6 (1.5);  C1: 5.8 (2.2); C2: 3.9 (1.8).  I no different to C1 (p=NS). I higher than C2 (p<0.05). | Not assessed. | **Knowledge:** Favours animation.  **Attitudes & Cognitions:**  No difference between animation and verbal consent.  Favours animation compared to a pamphlet. |
| Degirmentepe, 2025 (a), Turkey(27) | RCT | Female patients with urinary incontinence  Education level NR | Age I = 54.4 (8.8), C = 55.2 (5.4),  Male: 0% | N=70  I=35  Video animation + written and verbal information  C=35  Written and verbal information only | 2-minutes animated video which  provides detailed visual information about urodynamics using 3D animations. The participant is free to pause the video, rewind it, and ask any questions she wants.  Link:  https://patients.uroweb.org/videos/urodynamic -testing-video/ | Not assessed. | Satisfaction (VAS- 10-point Likert scale)  mean score (SD):  Post intervention:  I: 9.2 (1.1) (n=40);  C: 7.8 (1.3) (n=40);  p < 0.01. | Willingness to repeat the procedure (VAS-10-point Likert scale)  I: 3.4 (0.9) (n=40);  C:2.1 (1.3) (n=40);  p < 0.01. | **Attitudes and Cognitions:** Favours animation.  **Behaviours:** Favours animation. |
| Degirmentepe, 2025 (b), Turkey (26) | RCT | Patients scheduled for Extracorporeal Shock Wave Lithotripsy (ESWL)  Education level NR | Age  I = 41.0 (8.2)  C= 41.2 (7.5)  Male: 68% | N=80  I=40  Video animation + written and verbal information  C=40  Written and verbal information only | A short, 2.5-minutes video explains the ESWL procedure with 3D animation. A doctor accompanies the patient for real-time translation, allowing patients to rewind the video, ask questions, and get further explanations during the consent process.  Link:  Provided but not working. | Not assessed. | Satisfaction (VAS- 10-point Likert scale)  mean score (SD):  Post intervention:  I=8.8 (1.3) (n=40);  C=7.2 (2.0) (n=40);  p < 0.01, | Willingness to repeat the procedure (VAS-10-point Likert scale) :  I: 5.6 (2.0) (n=40);  C: 3.6 (1.9) (n=40);  p < 0.01. | **Attitudes and Cognitions:** Favours animation.  **Behaviours:** Favours animation. |
| Degirmentepe, 2025 (c), Turkey (28) | RCT | Patients undergoing flexible cystoscopy  Education level NR | Age  I=64.4 (8.8)  C=62.0 (10.4)  Male: 100% | N=160  I=80  Video animation + written and verbal information  C=80  Written and verbal information only | A video over 2 minutes long explains the cystoscopy procedure with 3D animation. Patients can rewind the video, ask questions, and seek further explanations if they did not understand or were confused about a point.  Link  Provided but not working. | Not assessed. | Satisfaction with the information (VAS- 10-point Likert scale)  mean score (SD):  Post intervention:  I = 9.2 (1.1) (n=80);  C = 8.6 (1.3) (n=80);  p < 0.01. | Willingness to repeat the procedure (Vas-10-point unit Likert scale):  I = 2.2 (0.4) (n=80);  C = 1.8 (0.3) (n=80);  p < 0.01. | **Attitudes and Cognitions:** Favours animation.  **Behaviours:** Favours animation. |
| Ellett, 2014, Australia(75) | RCT | Adults undergoing planned laparoscopy for pelvic pain.  Education level NR | Age: I:  39.8 (8.2); C: 32.3 (9.7).  Male: 0% | N= 41  I= 21 Multimedia including text, voice, and 3D animation + Usual care ( verbal consent with doctor)  C= 20  Usual care. | Multimedia information, a mixture of voice, text, photographs, and 3D computer animation, for operative laparoscopy lasting 15 mins.  Link NR. | Knowledge scale = 14 questions (score out of 14): mean (SE)  Stage 1 (straight after information delivery):  I: 11.3 (0.49) (n=21);  C: 7.9 (0.50) (n=20);  p<0.001.  Stage 2 (6 weeks later):  I: 8.4 (0.53) (n=19);  C: 7.8 (5.0) (n=17);  p=NS. | Satisfaction (recorded on 10cm VAS) mean (SD):  Post-intervention  I: 8.5 (1.9);  C: 8.2 (2.5); p=NS. |  | **Knowledge:** Favours animation at Stage 1 but no difference at Stage 2**.**  **Attitudes & Cognitions:** No difference between animation and verbal groups. |
| Friedman, 2025, Israel (96), | RCT | Women who  underwent at term Induction of labour (IOL)  Education level NR | Age:  I: 30.9 (5.6)  C: 30.1 (5.7);  Male: 0% | N=182  I= 91  Video animation + Usual care (standard counselling)  C=91  Usual care | Watched 8-mins animated video detailing IOL methods and risks in addition to standard counselling.  Watched it once.  Link NR. | Not assessed. | Satisfaction with procedure (possible range 1 to 5):  Post-intervention:  I: 4.4 (0.6) (n=81);  C: 4.1 (0.9) (n=80);  p=0.018. | Not assessed. | **Attitudes & Cognitions:** Favours animation. |
| Gois, 2024, Australia(95) | RCT | Patients undergoing clinically indicated percutaneous kidney  biopsies  No statistically significant difference in education level | Age: Median(range):  52 (IQR 34-65)  Male: 44% | N=124  I= 62  Video + Usual care (verbal information to inform consent)  C= 62  Usual care (verbal information to inform consent) | 8 minutes explanatory animation on an online platform  covering the procedure, its risks, and pre- and post-biopsy care before providing digital consent. The video allowed  participants to pause or rewind as needed.  Link NR. | Knowledge (possible range 0-9):  Median (IQR): Post-intervention  I: 8 (7 to 9) (n=60);  C:5 (4 to 7) (n=60);  p<0.001. | Satisfaction (scale of 1 to 7)  Median (IQR): Post intervention:  I:74 (69.8 to 77) (n=60);  C:76 (72.8 to 77) (n=60);  p=NS | Not assessed. | **Knowledge:**  Favours animation.  **Attitudes & Cognitions:**  No difference between arms. |
| Hermann, 2002, Austria (16) | RCT | Patients undergoing thyroid surgery  Education level NR | Age: 56.2 (NR)  Male: 22% | N= 80  I= 36  3D computer animation  C= 44  Written text | 7 minutes 3D animation which portrayed the process of thyroid surgery.  Watched once on the VHS video player.  Link NR. | Knowledge (of risks), mean score (SD).  I n=36; C n=44 for all knowledge outcomes.  Post-intervention:  I: 2.3 (1.2);  C: 2.2 (1.3);  p=NS.  Knowledge (of complications), mean score (SD):  I: 2.9 (SD NR);  C: 2.8 (SD NR);  p=NS. | Post-intervention:  Desire to know how the surgical procedure is carried out, means score out of 5 mean (SD).  I n=36; C n=44 for all attitude outcomes.:  I: 4.3 (1.2);  C: 3.7 (1.4);  p=NS.  Have better understanding of the surgical steps, mean score out of 5 (SD):  I: 4.5 (1.0);  C: 3.9 (1.2);  p=0.018.  Portrayal was lifelike, means score out of 5 (SD):  I: 4.6 (1.2);  C: 3.8 (1.2);  p=0.0008.  Fear of surgery was reduced, means score (SD):  I: 4.1 (1.1);  C: 3.0 (1.3);  p=0.00019.  Comfort with procedure (“the inner yes”), mean score (SD):  I: 4.5 (0.7);  C: 3.8 (SD NR);  p=0.004.  Professional competence, mean score (SD):  I: 4.9 (0.2);  C: 4.7 (0.7);  p=NS.  Would like to see real video of surgery, mean score (SD):  I: 2.4 (1.8);  C: 2.8 (1.8);  p=NS. |  | **Knowledge:** No difference between arms.  **Attitudes & Cognitions:**  Favours animation (4 out 7 items; no difference between arms on 3 out 7 items). |
| Homans, 2025, Netherlands (25) | RCT | Participants who were eligible for cochlear implantation  No statistically significant difference in education level | Age:  I= 68 (10);  C=68 (12);  Males=  I: 58%,  C: 56% | N=46  I=19  6 x 3D video animations + Usual care (standard cochlear implants selection process)  C= 27  Usual care | Participants received six additional 3D video animations (range 51 seconds to 23 mins, total 38 mins 51 secs) via email  alongside standard information. The videos were  accessible online through a link with no restrictions in the number of views.  Link:  https://tinyurl.com/CI-a nimations | Immediately after the intervention:  Self-assessment knowledge scores:  I: median 42 (n=27);  C: median 37 (n=19);  U = 156.5;  p= 0.025.  Objective knowledge scores:  I: median 84 (n=27);  C: median 72 (n=19);  U = 182.5;  p = NS. | Overall satisfaction (VAS scale):  I: median 9.5 (n=27);  C: median 8.6 (n=19);  U = 176.5,  p = NS. | Not assessed. | **Knowledge:** Favours animation for self-assessment knowledge;  no difference between arms for objective knowledge.  **Attitudes and Cognitions:**  No difference between arms. |
| Hong, 2012, Korea(17) | RCT | Emergency department patients about to undergo CT scan  No statistically significant difference in education level | Age: I= 38 (13.7); C= 42.3 (14.0),  Male:  I= 45%;  C= 56% | N= 150  I= 75  Animation + Usual care (verbal informed consent)  C= 75  Usual care | 7 minutes animation assisted informed consent about the contrast Computed Tomography (CT) scan.  Watched once on tablet computer.  Link NR. | Knowledge, combined mean score (SD).  I n=75; C n=75 for all knowledge outcomes.  Post intervention:  I: 8.6 (1.4);  C: 8.2 (1.5);  p=NS.  Individual knowledge items, mean score (SD):  Purpose:  I: 8.9 (1.5);  C: 8.3 (1.9);  p=0.026.  Process & method:  I: 8.9 (1.4);  C: 8.4 (1.8);  p=0.048.  Notice:  I: 8.8 (1.6);  C: 8.4 (1.8);  p=NS.  Complications:  I: 8.4 (1.8);  C: 8.2 (2.0);  p=NS.  Alternative:  I: 8.0 (2.2);  C: 7.4 (3.0);  p=NS. | Satisfaction, combined mean (SD).  I n=75; C n=75 for all satisfaction outcomes.  Post intervention:  I: 8.7 (1.3);  C: 8.1 (1.9);  p=0.045.  Individual satisfaction items, mean (SD):  Speed of explanation:  I: 8.6 (1.7);  C: 8.0 (2.1);  p=0.048.  Familiarity:  I: 8.8 (1.7);  C: 8.2 (2.1);  p= 0.048.  Overall satisfaction:  I: 8.8 (1.4);  C: 8.1 (2.2);  p=0.038. |  | **Knowledge (combined score):**  No difference between arms.  **Attitudes & Cognitions (satisfaction):** Favours animation**.** |
| Kakinuma 2011, Japan(37) | RCT | Patients about to undergo surgery for cancer.  No statistically significant difference in education level | Age: I= 60.1 (13.7), C= 60.0 (13.2),  Male: 59.2% | N= 211  I= 106  Cartoon animation + Usual care (visit from an anesthesiologist).  C= 105  Usual care. | 30 minutes (several brief sections which lasted 1 to 2 minutes) cartoon animation of the role of anaesthesiologists during surgery, gave nil per os (NPO) instructions, and the rationale for the NPO instructions, usual general or combined general and epidural anaesthesia procedures, and postoperative recovery.  Patients were allowed to repeat any part of the video as many times as they wanted within 30 minutes.  Watched on laptop, frequency NR.  Link NR. | Knowledge mean (SD):  After interview:  I: 12.5 (1.4) (n=106);  C: 11.2 (1.7) (n=105);  p< 0.0001. | Not assessed. | Not assessed. | **Knowledge:**  Favours animation. |
| Lattuca 2018, France(19) | RCT | Patients undergoing coronary angiography and/or angioplasty,  Cardiology units (39 participating centres in France)  No statistically significant difference in education level | Age: 67.3 (11.6),  Male: 72% | N= 843  I= 424  3D educational video + Usual care  C= 419  Usual care | 5 minutes 3D education video on the procedure of the coronary angiography  Watched once on a tablet.  Link:  <https://ars.els-cdn.com/content/image/1-s2.0-S0002870318300784-mmc1.mp4> | Knowledge, mean total score out of 16 (SD):  Post intervention:  I: 11.8 (2.8);  C: 9.5 (3.1);  p<0.001.  Knowledge, higher scores in I arm for all 6 sub-scale scores (all p<0.001). | Satisfaction with information, mean score out of 10 (SD):  Post intervention:  I: 8.4 (1.9) (n=406);  C: 7.7 (2.3) (n=415);  p<0.001. | Not assessed. | **Knowledge:** Favours animation**.**  **Attitudes & Cognitions:** Favours animation**.** |
| Lin, 2018, Taiwan(30) | RCT | Adults in Emergency Department due to have acute debridement surgery.  No statistically significant difference in education level | Mean ages not reported.  Male:  I=51%  C=60% | N = 142  I = 70. Animation about consent + Usual care (verbal information  C= 72  Usual care | 2D graphics animation, including 7 sections about the surgery and aftercare. Included subtitles and captions.  Length not reported.  Link NR. | Knowledge mean (SD):  Post intervention (immediately afterwards):  I: 72.6 (16.2) (n=70);  C: 61.7 (18.4) (n=72);  p<0.001. | Satisfaction with information. 3 questions, analysed individually. I n=70; C n=72.  Post intervention:  Comprehension: I > C p<0.001.  Helped me with decisions: I > C: p<0.001.  Satisfied with consent process: I > C: p<0.001. | Not assessed. | **Knowledge:** Favours animation**.**  **Attitudes & Cognitions:** Favours animation**.** |
| Lv, 2024, China(50) | RCT | Caregivers whose children underwent neurosurgical  Procedures  No statistically significant difference in education level | Age:  I: 34.4 (SD 7.8)  C: 34.4 (SD 8.9)  Male:  I= 36.8%  C= 34.1% | N=204  I=102  Animation assisted  nursing education + face‐to‐face oral nursing education  C=102  Face‐to‐face oral nursing education | Caregivers could watch the 4 minutes animated video repeatedly before the surgery and access it on their mobile devices any time after the session. The animation was available on the hospital's website when  using the local area network. | Knowledge (10‐item  Questionnaire) accuracy rate (%):  Post-surgery  I: 91.0% (n=87);  C: 82.0% (n=88);  p= 0.009. | Not assessed. | Not assessed. | **Knowledge:**  Favours animation. |
| Mayilvaganan, 2018, India(38) | RCT (3 Arms) | Patients who underwent hemithyroidectomy  Education level NR. | Age: Group 1 static images 31.2 (13.3), Group 2 3D model 35.4 (12.8), Group 3 Video 36.6 (11.8),  Male: 25% | N= 60  I1 (Group 3)= 20  Animated video  I2 (Group 2) = 20  3D thyroid model  C (Group 1) = 20  Conventional diagram | 2 minutes animated cartoon video of the hemithyroidectomy procedure  Animation frequency and delivery NR.  Link:  IJEM-22-520-v001.mp4 | Not assessed. | Patient Satisfaction Questionnaire**:**  Post intervention:  I n=20; I2 n=20; C n=20.  Experience with the model, mean (SD):  Group 1: 1.8 (0.4);  Group 2: 1.7 (0.6);  Group 3: 2.5 (0.4);  p<0.01.  Overall satisfaction, mean (SD):  Group 1: 2.1 (1.1);  Group 2: 1.9 (1.0);  Group 3: 2.0 (0.7);  p=NS.  Unmet informational needs were met:  Group 1: 50% patients;  Group 2: 65% patients;  Group 3: 75% patients.    Group 3 vs Group 1: p=0.12.  Group 3 vs Group 2:  p=NS. | Not assessed. | **Attitudes & Cognitions:** Favours animation on one item.  No differences between groups on other items or overall score. |
| Mednick 2016, Canada(68) | RCT (3 Arms) | Patients undergoing an initial intravenous fluorescein angiography (IVFA) investigation  Education level NR | Age: 66.9 (12.4),  Male:56% | N= 78  I= 26  Narrated white board animation    C1= 26  Usual care(Standard consent)  C2= 26  Narrated white board animation + Usual care | 3.5 minutes narrated whiteboard animation of the procedure of intravenous fluorescein angiography (IVFA).  Watched on computer, frequency NR.  Link NR. | Knowledge, mean score out of 6 (SD): Post intervention:  All outcomes I n=26; C1 n=26; C2 n=26.  I: 5.0 (0.9)  C1: 4.4 (1.1)  C2: 5.6 (0.6)  I vs C1: p=0.023;  C2 vs C1: p<0.001;  C2 vs I: p=NS. | Satisfaction:  3 questions:  Post intervention:  (1) fully understood what IVFA entailed; (2) felt well informed on consent; (3) understood the thoroughness of consent process:  I vs C1 and C2 vs C1; all 3 p=NS. | Not assessed. | **Knowledge:**  Favours animation, and combined animation + Usual care.  **Attitudes and Cognitions:**  No differences between arms. |
| Mhalu 2015, Tanzania(64) | RCT | Presumptive tuberculosis infection (TB) patients, Outpatient department in Tanzania  Education level NR | Age: Median (range): 39.1 (37.0 to 50.0);  Male: 53% | N= 200  I= 100  Video animation    C= 100  Usual care (verbal instructions) | 4 minutes animated sputum submission instructional video .  Watched once on a laptop.  Link:  <https://www.youtube.com/watch?v=2sd2d2_pNBA> | Not assessed. | Not assessed. | Quality of sputum sample:  Post intervention:  All behaviour outcomes: I n=100; C n=100.  I: %NR;  C: %NR;  p<0.0001.  Sputum volume adequate:  I: 78%;  C: 45%;  p<0.0001.  Saliva sample provided (i.e. incorrectly):  I: 14%;  C: 39%;  p=0.0001. | **Behaviours:** Favours animation. |
| Miao 2020, Australia(78) | RCT | Patients referred for Mohs micrographic surgery, 2 large outpatient dermatology facilities in Sydney, Australia  No difference in education level | Age: I= 63.1 (10.9), C= 65.1 (13.0),  Male: 55% | N=102  I= 51  Video + Usual care (verbal consent)  C= 51  Usual care | 5.50 minutes video animation which outlined the standard consent for Mohs micrographic surgery. The video was designed to supplement rather than replace the patient’s consultation with his or her physician.  Watched on iPad, Frequency NR.  Link NR. | Knowledge, means score out of 10, % (SD):  Post intervention:  I: 8.6% (1.8) (n=51);  C: 6.3% (2.6) (n=50);  p=0.02.  The intervention arm had higher knowledge scores on 8/10 individual items (at p<0.05). No difference between arms on 2/10 items (p=NS). | Satisfaction, total mean score out of 35 (SD):  Post intervention:  I: 31.5 (3.1) (n=51);  C: 30.1 (4.4) (n=49);  p=NS.  The intervention arm had higher satisfaction scores on 1/7 items (p=NS) with no difference between arms on 6/7 items (p=NS). | Not assessed. | **Knowledge(over all score):** Favours animation.  **Attitudes & Cognitions:**  No difference between arms. |
| Mladenovski 2008, New Zealand(76) | RCT | Patients referred to the University of Otago School of Dentistry for third molar extraction  Education level NR | Age (range): 16 to 49yrs  Male: 25% | N= 30  I= 16  Multimedia  C= 14  Leaflet | 6 minutes animation of the third molar extraction 3D programme. The multimedia software employed audio and visual files on a CD-ROM.  Watched on computer.  Link NR. | All outcomes: I n=16; C n=14.  Knowledge, 14 items:  Post intervention:  No differences between arms on any of the 14 items (all p=NS). | Self-rated knowledge:  % rated themselves as ‘excellent or very good or good’:  Post intervention:  I: 62.5%;  C: 38.5%;  p=NS.  Satisfaction ratings of intervention, 12 items, (% agreeing):  ‘Helpful’  I: 93.8%;  C: 15.4%;  p<0.05.  ‘Preferred’  I: 75.0%;  C: 15.4%;  p<0.05.  No difference between arms on other 10/12 items. | Not assessed. | **Knowledge:** No difference between arms.  **Attitudes & Cognitions:**  Self-rated knowledge no difference between arms;  Satisfaction: favours animation on 2/12 items; no difference between arms on 10/12 items. |
| Mofrad 2021, Netherlands (88) | RCT (3 arms) | Patients visiting the memory clinic of the Alzheimer Centre Amsterdam.  No statistically significant difference in education level between the 3 groups | Age: 63 (9)    Male:  I1: 53%; I2: 69%; C: 70% | N= 209  I1= 63  Animation viewing at home + Usual care (verbal information and an informational folder on the LP procedure)  I2 =70  Animation viewing in clinic + Usual care  C= 76  Usual care | 3 minutes animation video to inform and prepare patients and caregivers for the Lumbar puncture (LP) procedure in the context of Alzheimer’s Disease (AD) diagnosis which included the LP procedure and the most common complications.  All participants also received care as usual.  Home viewing group were allowed to watch the video as often as desired. Clinic viewing group viewed the video once in the waiting room.  Link NR. | Information recall:  T2 (screening day)  I1 vs C:  mean difference 0.97 (0.29) (I1 n=62; C n=74) (p=0.003).  I2 vs C:  mean difference 1.35 (0.28) (I2 n=67; C n=74) (p<0.001).  T3 (end of screening day)  I1 vs C:  mean difference 0.84 (0.26) (I1 n=62; C n=74); (p=0.005).  I2 vs C:  mean difference 0.9 (0.26); (I2 n=67; C n=74); (p=0.002).  There was no difference in information recall between home and clinic viewing at either T2 or T3. | Satisfaction: no difference in satisfaction between interventions and control in terms of satisfactory F [2,176] = 2.24,  (p=NS).  (I1 n=61; I2 n=67; C n=74) | Not assessed. | **Knowledge:**  Favours animation.  **Attitudes & cognitions**  No difference in satisfaction**.** |
| Molher, 2022, France(79) | RCT | Adults due to have planned surgery for benign parotid tumour.  No difference in education level | Age mean (range):  I:54 ( 27-85);  C: 50 ( 20-87)  Male: I: 52%;  C: 55% | N= 69  I= 34  Animation + written information + usual care (verbal information)  C = 35  Written information + usual care. | 15 minutes animation bespoke for the study. It includes all the info in the written information. It included info on 7 postoperative risks.  Watched unlimited on smartphone.  Link NR. | Number of risks of surgery recalled by participants (in response to single open question) median (IQR).  Post intervention (eve of surgery):  I: 2 (2-3) (n=27);  C: 1 (0-2) (n=29(;  p<0.005. | Not assessed. | Not assessed. | **Knowledge:** Favours animation**.** |
| Moore, 2020, USA(52) | RCT | Adult patients undergoing BRAVO placement for gastric reflux  Education level NR | Age: 54(17)  Male: 40% | N=120  I= 60  Animation + Usual care (two pages of written information)  C= 60  Usual care. | 4-minute animated  instructional video freely accessible on YouTube distributed by the clinical team.  Link:  https://www.youtube.com/watch?v=oHblYttKHGw | Patient comprehension (completion of periprocedural tasks):  Post intervention:  I n=44; C n=34.  Means not stated.  ANOVA I > C (F = 9.14; p=0.003) on comprehension. | Satisfaction  (5-point Likert scale)  Post intervention:  I n=44; C n=34.  Means not stated.  ANOVA I > C (F = 44.1;  p<0.001). | Overall compliance with advice post-procedure:  I n=44; C n=34.  I: 9.6 (SD 1.4);  C: 7.4 (2.0);  p= 0.01 | **Knowledge:** Favours animation**.**  **Attitudes and Cognitions:** Favours animation**.**  **Behaviour:**  Favours animation. |
| Pallett, 2018, USA(80) | RCT | Women undergoing planned hysterectomy (for benign condition)  No difference in education level | Age:  I: 41.4;  C: 44.2  Both groups males = 0% | N = 120  I= 60  Video including 'diagrams, illustrations, and animations' + Usual care(standard physician counselling).  C=60  Usual care. | 10 minutes video including 'diagrams, illustrations, and animations'.  Watched on tablet, only once.  Link NR. | All outcomes: I n=57; C n=52.  Knowledge:  Immediately post-intervention:  I: 81.5%;  C: 67.5%;  p =0.0009.  Day of surgery:  I: 74.0%;  C: 64.5%;  p=0.02.  6 weeks post-surgery:  I: 66.0%;  C: 62.5%;  p=NS. | Satisfaction: (assessed immediately after intervention) mean:  I: 30.9;  C: 30.9;  p=NS. | Not assessed. | **Knowledge:** Favours animation on 2/3 assessments. (1/3 no difference between groups).  **Attitudes & Cognitions:** No difference between groups. |
| Platto 2019, USA(29) | RCT | Patients awaiting dermatologic surgery  Education level NR | NR | N= 45  I= 22  Video animation + Usual care(conventional consultation with the surgeon)  C= 23  Usual care. | 2 minutes animated educational video covering anaesthesia, excision, repair, post-operative wound care, and pain management  Frequency and delivery NR.  Link:  <https://www.youtube.com/watch?v=x7ujWviMNxM> | Not assessed. | Want additional information about procedure, mean (SD):  Post intervention:  I: 5.1 (2.0) (n=22);  C: 6.3 (3.1) (n=23);  p=NS. | Not assessed. | **Attitudes & Cognitions:** Desire for more information: No difference between arms. |
| Reynolds-Wright 2020, UK (63) | RCT and Quasi RCT Paris site | Gynaecological patients with confirmed gestation in abortion clinics in three locations: Paris, Stockholm and Edinburgh  Education level NR | Age: I= 28 (NR); C= 27.8 (NR)  Male: 0% | N= 172  I=104  Animation  C= 68  Standard care (face-to-face consultation) | 3 minutes video animation summarised the process of early medical abortion (EMA) using simple language and animated characters representing women of diverse ages and ethnicities.  Watched once on a laptop computer in a private room in the clinic.  Link:  <https://vimeo.com/302663935> | Knowledge, mean score out of 8 (SD):  Post intervention:  Site 1:  I: 3.5 (n=35);  C: 3.3 (n=15);  p=NS.  Site 2:  I: 3.9 (n=35);  C: 2.9 (n=39);  p=0.007.  Site 3:  I: 5.3 (n=34);  C: 5.1 (n=14);  p=NS.  Combined sites:  I: 4.2 (0.4) (n=104);  C: 3.5 (3.9) (n=68);  p<.05 | Post intervention: Information :  ‘very helpful’: Site 1:  I: 97%;  C: 93%;  p=NS.  Site 2:  I: 74%;  C: 100%;  p=0.001.  Site 3:  I: 79%;  C: 64%;  p=NS.  Information ‘very clear’:  Site 1:  I: 91%;  C: 93%;  p=NS.  Site 2:  I: 89%;  C: 77%;  p=NS.  Site 3:  I: 79%;  C: 64%;  p=NS.  Information utility (rated 10/10):  Site 1:  I: 76%;  C: 60%;  p=NS.  Site 2:  I: 86%;  C: 67%;  p=0.048.  Site 3:  I: 68%;  C: 64%;  p=NS. | Not assessed. | **Knowledge:** Favours animation.  **Attitudes & Cognitions:**  Favours standard care at 1/3 sites on information ‘very helpful’. No difference between arms at 2/3 sites.  No difference between arms on information ‘very clear’ at all 3 sites.  Favours animation on utility at 1/3 sites. No difference between arms at 2/3 sites. |
| Roy, 2025, Canada(51) | RCT | Children undergoing adenotonsillectomy  No statistically significant difference in caregiver education level | Age: 5.2 (3.0)  Male: 55%  NB outcome measures were completed by caregivers (age and sex not reported). | N=142  I=71  I= Animated audiovisual + Clinician led teaching + pamphlet  C=71  Clinician led teaching + pamphlet | Patients and  caregivers were given the option to view the platform unlimited times on a  clinic computer, or via any web‐based platform of their  choice via a link sent by electronic mail.  Link: precare.ca/healthcare‐guides/tonsillectomy/ | Not assessed. | Caregiver satisfaction using the Care Transitions Measures (CTM-15) questionnaire  Post-operation:  mean (SD):  I: 85.8  (12.9) (n=50);  C: 85.4  (16.2) (n=50);  p=NS. | Unplanned Postoperative Medical Attention:  I=13 (26%) (n=50);  C=15 (30%) (n=50);  p=0.66. | **Attitude and Cognitions:**  No difference between arms.  **Behaviours:** no difference between arms. |
| Sahebalam, 2020, Iran (62) | RCT | Healthy paediatric dental participants in the clinical department of pediatric dentistry in Iran  Similar level, all in Primary school | Age: 5.23 (0.63)  Male: 35.4% | N= 50  I= 25  Video animation  C= 25  Tell-Show-Do technique | 1.14 minutes animation called: Jilo goes to a dentist. Dental instruments and procedures are designed in the form of objects and concepts that a child can understand, and that involve their imagination. For example, the suction procedure has been modelled using an elephant's trunk and its power to suck in water.  Watched once.  Link:  <https://www.aparat.com/v/g7wFt> | Not assessed. | Not assessed. | Non-cooperation at 1^st^ visit (30 mins post intervention, mean score out of 5) (SD):  I: 0.3 (0.6) (n=24);  C: 1.0 (1.1) (n=24);  p=0.015.  Non-cooperation at 2^nd^ visit (a week after), mean score out of 5) (SD):  I: 0.6 (0.9) (n=24);  C: 1.3 (1.1) (n=24);  p=0.019. | **Behaviour:** Favours animation. |
| Sariturk, 2017, Turkey (81) | RCT | Adult patients who were scheduled to undergo autologous or allogeneic peripheral stem cell transplantation (SCT) at the Adana Bone Marrow Transplantation Unit of Baskent University Faculty of Medicine and donors from whom peripheral stem cell collection for allogeneic transplantation was planned  No statistically significant difference in education level | Age: 47 (14)  Male: 56.1% | N= 82  I= 42  Audio-visual + Usual care ( verbal + written information)  C=40  Usual care (verbal + written information) | 10 minutes information animation covered disease status, purpose of the treatment, treatment principles, stem cell collection procedure, pre-treatment assessment, the drugs used and their side effects, infusion of stem cells, benefits expected from the treatment, treatment risks and side effects, other treatment options, and disposal of the cellular product.  Watched once.  Link NR. | Not assessed. | I n=42; C n=40 for all outcomes.  Satisfaction, overall mean out of 200 (SD):  I: 185.7 (22.2);  C: 183.8 (17.2);  p=NS.  Satisfaction with written informed consent sub-score, mean score out of 70 (SD):  I: 64.2 (8.5);  C: 60.3 (8.2);  p=0.039.  Satisfaction with informing doctor sub-score out of 70 (SD):  I: 64.7 (8.6);  C: 67.0 (5.8);  p=NS. | Not assessed. | **Attitudes & Cognitions:** Overall satisfaction, no difference between arms.  One sub-score favours animation; one sub-score no difference between arms. |
| Shi, 2025, China(34) | RCT | Patients with atrial fibrillation (AF) undergoing atrial fibrillation catheter ablation  No statistically significant difference in education level | Age:  I: 61.2 (10.9)  C: 60.7 (11.3)  Male:  67.3% | N=226  I=113  Digital animation + Usual care  C=113  Usual care | Digital animation-based multistage education programme, which  is categorised as routine and non-invasive patient education lasted < 5 mins.  Link NR. | Not assessed. | AF Quality of Life measure (AF-QOL-18) score:  3 months post intervention:  I: 47.8 (5.7) (n=104);  C:45.3 (5.7) (n=104);  p<0.001. | Medication Adherence Report Scale (MARS-5): mean (SD)  3 months post discharge:  I: 20.1 (2.1) (n=104);  C: 18.5 (2.8) (n=104); p<0.001. | **Attitudes & Cognitions:** Favours animation.  **Behaviour:**  Favours animation. |
| Shqaidef 2021, Jordan(20) | RCT | Adolescents undergoing first orthodontic treatment.  Education levels similar in 2 groups. | Age:  I: 14.0;  C: 14.6  Male:  I: 47% ;  C: 44% | N = 64  I=32; 3D animation.  C=32; Orthodontics leaflet (given 10 minutes to read it). | 3D cartoon animation, watched it on a tablet, watched once only.  Length not reported.  Link NR | Knowledge: 13 open-ended questions score total out of 25 (adjusted to give score out of 100) mean (SD).  1 year post-consent:  I: 74.4 (9.0) (n=31);  C: 75.6 (12.3) (n=31);  p=NS. | Not assessed. | Not assessed. | **Knowledge:** No difference between animation and written information groups. |
| Tipotsch-2016, Austria (21) | RCT | Patients who were scheduled for surgery for age-related cataract in Hietzing Hospital, Vienna  Education level NR | Age: 71 (7)  Male: 41% | N= 123  I=59  Computer animated video + face-to-face consultation + brochure  C= 64  Face-to-face consultation + brochure | 6 minutes computer-animated video (Eyemaginations 3D-Eye, Eyemaginations) of the cataract surgery which covers background information, surgery, complications, post-op therapy and controls  Frequency and delivery NR.  Link NR. | I n=59; C n=64 for all outcomes.  Knowledge, mean score out of 10 (SD)(overall mean score):  Immediately after the consent procedure:  I: 8.2 (0.5);  C: 7.2 (0.7);  p=0.002.  Animation arm had higher score on 4/10 items (p<.05) with no difference between arms on other 6/10 items. | Satisfaction with consent, mean score out of 5 (SD):  Immediately after the consent procedure:  I: 1.1 (0.4);  C: 1.0 (0.2);  p=NS.  (low score = more satisfied) | Not assessed | **Knowledge:** Overall knowledge score favours animation.  **Attitudes & Cognitions:** No difference between arms. |
| Tou, 2013, Australia (31) | RCT | Patients due to have planned bowel surgery, colorectal Unit at the Queen Elizabeth Hospital, Adelaide, Australia  Education level NR | Age: 59 (31)  Male: 39% | N= 31  I=16  Cartoon animated video + information sheet  C=15  Information sheet alone | 13 minutes 2D cartoon animation about bowel surgery including pre-, peri- and postoperative care.  Watched the animation film on a desktop computer in a quiet room, frequency NR.  Link NR. | Knowledge, mean score (SD):  Post intervention:  I: 5.8 (0.5) (n=16);  C: 5.8 (0.6) (n=16);  p=NS. | Not assessed. | Not assessed. | **Knowledge**: No difference between arms. |
| Tucker, 2022, USA(73) | RCT | Adults due to have planned endometrial surgery for cancer staging.  P-value is not reported. The intervention group has higher educational degrees; however, when stratified by whether patients have at least some college or a greater degree, no differences are noted between the two groups. | Mean ages NR but distribution in both groups looks similar.  Both groups 0% male. | N=80  I = 40; Multimedia education tool (2 videos): one on the surgical procedure and one on the consent process. Cartoon + 3D animations. Followed by Usual care (physician education about surgery)  C = 40 Usual care | Two animations. (i) on surgery, length 4 minutes 55 seconds. 3D anatomical animation.  (ii) static cartoons and script. Length 3 minutes 45 seconds.  Videos viewed once only before standard physician education.  Links NR. | 9-item comprehension survey (designed for the study), scored 0-100. Higher scores = more knowledge. Time 1: enrolment. Time 2: pre-surgery or within 24 hours of surgery. Time 3: at clinic 4-6 weeks after surgery.  Time 1 (Pre-op):  I: 83.3 (95% CIs 78.6 – 88.0) (n=36);  C: 77.9 (73.3-82.4) (n=36);  p=NS.  Time 2 (Peri-op):  I: 81.0 (76.6-85.5) (n=32);  C: 81.4 (76.4-86.4) (n=32);  p=NS.  Time 3(Post-op):  I: 80.7 (75.1-86.2) (n=31);  C: 78.3 (72.7-83.9) (n=31);  p=NS. | Satisfaction Pre-op visist:  :  CSQ-8 (8 items). Scores range 8-32. Higher scores more satisfaction.  I: 31.7 (31.5-31.9) (n=36);  C: 30.7 (30.0-31.4) (n=36);  p<0.01.  Global satisfaction rating (1-10) mean (95%CI):  I: 9.95 (9.87-10.02) (n=36);  C: 9.74 (9.55-9.92) (n=36);  p=0.04. | Visit length (for pre-op education):  I: 90.36 minutes (95% CI 79.37-101.36) (n=36);  C: 80.46 (95% CI 71.94-88.99) (n=36); p=0.04. | **Knowledge:** No difference between arms.  **Attitudes & Cognitions:** Favours animation.  **Behaviour:** Favours control. |
| Turkdogan, 2021, Canada(82) | RCT | Adults due to have planned head and neck surgery.  Education: graduates. Int: 54.1%; C: 50%. | Age: Mean ages not reported.  Male:  I: 50.8%;  C: 45% | N = 121  I=61  ‘Precare’ animation + Usual care (in-person preoperative education)  C=60  Usual care | Precare (featuring prep for upcoming surgery; what to expect; steps to help with recovery in hospital and at home). Used 6th grade literacy level. Available in French and English, plus subtitles into 20 languages, plus their standard oral information.  Available in French and English languages, and included subtitles.  11-12 minutes long.  Viewed in clinic (if wanted) and were able to view it at home.  Link NR. | Not assessed. | I n=50; C n=50 on all outcomes.  Satisfaction:  (cumulative scores on postop Q)  I vs C=72.4 vs 61.1 (mean difference 11.3, 95% CI, 10.1 to 12.5; Cohen d = 1.02);  p<0.05.  Attitudes & Cognit Service information:  I vs C =169 vs 131 (mean difference 64, 95% CI 60.2 to 68.9; Cohen d =1.28);  p<0.05.  Treatment Information:  I vs C = 195 vs 143 (mean difference 52 (95% CI 44.5 to 59.2; Cohen’s d =1.05);  p<0.05.  Medical Tests Information:  I vs C = 174 vs 129 (mean difference 45 (95% CI 40.1 to 49; Cohen’s d =1.05);  p<0.05. | Not assessed. | **Attitudes & Cognitions:**  Favours animation. |
| Winter 2016, Australia(39) | RCT | Patients with acute renal renal colic to a public hospital (two teaching hosptials in Australia) who required a ureteric stent.  No statistical significant difference in education level | Age: 54 (NR)  Male: 75% | N= 88  I=43  Audio-visual presentation with cartoon animation  C=45  Standard verbal consent | 7:07 minutes cartoon visual animation regarding the consent process for cystoscopy and insertion of ureteric stent.  Watched on iPad, Frequency NR.  Link:  <https://bjui-journals.onlinelibrary.wiley.com/doi/10.1111/bju.13595> | Knowledge, mean score (95% CI) out of 28:  Post intervention:  I: 23.3 (22.2-24.3) (n=43);  C: 20.1 (18.6-21.6) (n=45);  p<0.001. | Satisfaction with care, mean score (95% CI) out of 32:  Post intervention (crossed over from standard verbal to animation):  I: 30.2 (29.4-31.0) (n=43);  C: 29.1 (28.0-30.2) (n=45);  p=NS. | Not assessed. | **Knowledge:** Favours animation.  **Attitudes & Cognitions: satisfaction** No difference between arms. |
| Yap, 2020, Singapore (71) | RCT (3:1) | Patients undergoing coronary angiography and/or angio plasty, recruited from tertiary cardiac institutions.  The intervention group had significantly lower education levels compared with the control group (p = .024) | Age: 59.0 (94)  Male: 86% | N= 332  I= 252  Video + Usual care (counselling by the physician +information sheet)  C=80  Usual care | 3 minutes whiteboard animated patient education video of the coronary angiography and/or angioplasty procedure.  Watched on smart phones, tablets as well as computers  Frequency NR.  Link:  <https://youtu.be/R8AdaIbNq7Y> | Knowledge, mean score out of 12 (SD):  I: 10.2 (1.7) (n=252);  C: 8.5 (2.9) (n=80);  p<0.001. | Not assessed. | Not assessed. | **Knowledge:** Favours animation. |

I= Intervention, C= Control, NR=Not reported, NS= Not significant, RCT=Randomised Controlled Trial
