# Supplementary material for: How effective are video animations as information tools for patients and the general public? An updated systematic review
Source: Front Digit Health. 2026 Jan 2;7:1717044. doi: 10.3389/fdgth.2025.1717044 (PMC12808424; doi:10.3389/fdgth.2025.1717044)
Supplement: Supplementary file 4 [file Table3.docx]

### Table 6: Category 2 - Management of health conditions (24 trials)

| **Author, year, country** | **Study design** | **Participants, Setting, Education level between the 2 groups** | **Age Mean (SD)/ % Male** | **Total sample;**  **Intervention descriptor & sample size (I);**  **Control descriptor & sample size (C)** | **Intervention details and link(if provided)** | **Results (Intervention vs Control)** (**Knowledge = knowledge or understanding. Attitudes & Cognitions = satisfaction or self-efficacy or confidence in decision, etc. Behaviours = behaviours or skills or intended behaviours), p value**  | | | | **Group favoured** | |
| --- | --- | --- | --- | --- | --- | --- | --- | --- | --- | --- | --- |
|  |  |  |  |  |  | **Knowledge** | | **Attitudes & Cognitions** | **Behaviours** |  |  |
| Akca Sumengen 2023, Turkey(42) | RCT | Children aged 7-11 with chronic allergic asthma, in university hospital in Istanbul.  Education levels are similar. | Ages:  I: <9 44.7%; 9+ 55.3%;  C: <9 44.4%; C:9+ 55.6%  Male:  I=60.5%  C=58.3% | N = 74  I=38  cartoon animation on asthma + colour-in materials + standard education information from doctors  C=36  Standard education information from doctors | Health promotion programme for children with asthma. (which included topics on pathology, symptoms, medications, inhaler techniques). PowerPoint slides followed by cartoon episode (plus child given comic to colour in related to that episode).  Watched once.  Link NR but source paper is cited. | Not assessed. | | I n=38; C n=36 for all outcomes.  Quality of life (PAQLQ, possible range 23-161) mean (SD):  T2: One month post-intervention: I: 91.4 (12.8); C: 73.3 (11.1); p<0.001.  T3 (4 months post-intervention):I: 114.2 (12.7); C: 78.2 (16.6); p<0.001. | School absenteeism (any, scored yes)  1^st^ month post-intervention:  I: 26.3%;  C: 72.2%; p<0.001. | **Attitudes & Cognitions:**  Favours animation.  **Behaviour:** Favours animation. | |
| Baker 2018, USA (83) | RCT | Patients undergoing anorectal testing for chronic constipation.  No statistical significant difference in education level | Age: 47.3 (16)  Male: 16% | N= 100  I= 50  Animated educational video  C= 50  Traditional written educational pamphlet | 2 minutes animated videos (3 short videos about improving chronic constipation.  Watched once on PC.  Link:  <https://mygi.health/education/symptoms/constipation> | Knowledge, mean score out of 13 (SD):  I: 11.2 (1.8) (n=50);  C: 11.1 (1.8) (n=50);  p=NS. | |  |  | **Knowledge:** No difference between interventions. | |
| Calderon 2014, (60) USA | RCT | Latino/Hispanic patients who were diagnosed with Type 2 diabetes at South Central Family Health Center (SCFHC) and the Charles Drew University of Medicine and Science Center for Health Services Research (Drew).  No statistically significant difference in education level | Age (Range) : 18 to >60  Male: 18.3% | N= 240    I= 118 Animated video  C= 122 Control (easy-to read text) | 13 minutes animated video which featured an animated icon named “Corazón Quelate” (heart that beats; Spanish version) / “Lotta Hart” (English version). Corazón/Lotta engages viewers with an invitation into her home and emphatically shares her experience with diabetes.  Frequency NR.  Link NR. | Change in adjusted DHLS (about diabetes) mean (%): 113 (55%) (n=113) vs 109 (53%) (n=109), F = 4.7, df = 1, p =0.03  Change in DHLS score in participants with inadequate functional health literacy (STOFHLA scores < 17) mean (%):  61 (53%) vs 52 (50%); F = 7.12, p = 0.009.  Change in DHLS score in participants with marginal or adequate functional health literacy (STOFHLA scores ≥ 17 mean (%): 37 (58%) and 50 (57%), F = 0.82, p =NS. | |  |  | **Knowledge**:  Favours animation overall, in particular  in participants with inadequate functional health literacy.  No difference in participants with marginal or adequate functional health literacy. | |
| Chakravarthy 2018, USA (70) | RCT | Patients prescribed opioids, emergency department of a large, urban academic hospital in USA.  Education level NR | Age: I=41 (NR); C=34 (NR)  Male: NR | N= 52  I= 25  Video discharge instruction on opioid safety and proper usage, storage, and disposal + Usual care  C= 27  Usual care | 6-minute animated video on proper usage of opioids in addition to standard of care.  Frequency NR.  Link:  https://youtu.be/5FYNBvgmdsE | Knowledge acquisition of opioid education [correct answer out of 26, mean (SD)]:  21.2 (4.98) (n=27) vs 16.8 (4.53) (n=26), p=0.001, Cohen's d= 0.92. | |  |  | **Knowledge:** Favours animation. | |
| Cleeren 2014, Belgium (22) | RCT | Adult patients with periodontitis, dental practice located in Kester, Belgium.  No statistically significant difference in education level | Age: 54.4(10.3)  Male: 29% | N= 67  I= 33  3D animation giving a general view of periodontitis  C= 34  Real-time sketches of a general view of periodontitis | 6.20 minutes 3 D animated video about the general view of periodontitis beginning with the periodontal anatomy followed by the causes, symptoms, development and treatment of the disease.  Patients were allowed to pause and rewind the videos on the computer screen during the clinic visit.  Link NR. | I n=33; C n=34 for all outcomes.  Knowledge on periodontitis mean (SD):  post-test:  I: 8.42 (1.1);  C: 7.1 (1.6);  p<.0001.  2 weeks follow up:  I: 7.5 (1.0);  C: 4.9 (1.3);  p<0.001.  Knowledge recall (mean difference from baseline):  post-test:  -0.4 vs -1.3 (p<0.001).  2 weeks follow up: 1.0 vs 2.2 (p=0.03). | |  |  | **Knowledge:** Favours animation. | |
| Diniz, 2022, Brazil (59) | RCT (3 arms) | Adults with non-specific low back pain attending outpatient physiotherapy clinics.  No statistically significant difference in education level | Age (mean, SD):  I: 48.9 (16.7);  C1: 51.5 (17.9);  C2: 48.3 (19.4)  Male:  I: 47.2%;  C1: 47.2%;  C2: 38.5% | N = 159  I= 53  Video animation  C1=53 Infographic  C2= 53 Written information | The video  animation used storytelling elements, including a story of a character who devel-  oped low back pain after carrying a box in the  garage. Then, two possible scenarios are contrasted.  during the story, one management strategy that  follows clinical practise guideline recommenda-  tions versus one based on low-value care.  Length 5 minutes. Watched only once.  Link:  https://youtu.be/  HvcqG3dfp8A | n =53 in each of the three trial arms on all outcomes.  Knowledge:  Q1 (on use of X-rays or scans):  Q2 (on need for X-ray or CT or MRI):  Incorrect beliefs:  I:29(54.7%);  C1:8(15.1%);  C2:13(24.5%);  Disagree with imaging prescription, lower score better outcome (5 point Likert scale) median (IQR):  I:3(2-4);  C1:2(1-2);  C2: 2(1-2);  Video knowledge improved less well than in the infographic group, and less well than the written summary group. | | Back Beliefs (scale scored 9-45) mean (SD):  I: 36.5 (5.8);  C1: 35.9 (4.7);  C2: 35.8 (5.2);  I vs C1  (p=0.34);  I vs C2 (p=0.2). | Not assessed. | **Knowledge:** Favours written summary and infographic**.**  **Attitudes & Cognitions:** No difference between animation, and infographic, and between animation and written summary. | |
| Di Pietro, 2024, Italy (23) | RCT | Adult patients with atrial fibrillation (AF) or deep vein thrombosis (DVT), being discharged home from emergency department.  Participants in the intervention group had a higher level of education, p value was not reported | Age. I: 68.5 (13.9);  C: 68.6 (14.0)  Male:  I: 47.2%; C: 55.6% | N= 144  I= 72  Video animation + Usual care  C= 72  Usual care | Video clip related to either AF or DVT. 3D animation clip, without audio but explained by the doctor as they were watching it.  27-45 seconds long.  Watched only once.  Link NR. | I n=72; C n=72 for all outcomes.  Knowledge of AF or DVT mean (95% CI):  48 hours post-discharge  Knowledge of diagnosis & possible complications (possible range 0-18):  I: 8.3 (7.3 – 9.3);  C: 5.9 (5.0 – 6.7);  p<0.001.  Knowledge of prescribed therapy (possible range 0-6):  I: 3.2 (2.7- 3.7);  C: 3.0 (2.6 – 3.4);  p=NS. | | Satisfaction with information mean (95% CI)  48 hours post-discharge  Satisfaction with information (possible range 0-12):  I: 8.0 (7.2 – 8.8);  C: 7.3 (6.4-8.2);  p=NS. | Initiation rate of newly prescribed anitcoagulants:  (48 hours after discharge):  I: 46/51 (90.2%)  C: 36/45 (80%)  OR = 2.3,p=NS. | **Knowledge:** (measure i) Favours animation and (measure ii) no difference between arms.  **Attitudes and Cognitions:** No difference.  **Behaviour:** No difference. | |
| Flynn, 2023, USA (84) | RCT | Pregnant women with risk of pre-term birth.  No statistically significant difference in education level | Mean ages NR.  Male: 0% in each group | N= 120  I= 60  Video animation pack (51 videos), prompted to watch them by daily text messages  C= 60  Given links to patient education webpages (created by American College of Obs & Gynae), prompted by email or text message | I: 51 videos (length 1-3 minutes), which were specific to the gestational age.  Videos viewed by 39.8% to 61.4% of Int participants.  Link NR. | PPKQ (Parent Prematurity Knowledge Questionnaire), 10 items, scored 0-100:  Timing mean (SD): T1- 25 weeks gestational age; T2- 30 weeks gestational age; T3- 34 weeks gestational age (only taken if participant still pregnant).  I:  T1: 79.8 (SD 5.8); n=54;  T2 86.0 (5.1); n=52;  T3: 8 5.9 (5.6); n=54.  C:  T1: 59.9 (5.8); n=53;  T2: 70.4 (5.1); n=52;  T3: 71.0 (5.6); n=49.  I vs C  T1: p<0.001  T2 and T3 p values not reported but were both at least p<0.05 (can be calculated). | | Preparation for Decision Making Scale. (10 items) mean scored 0-100. Higher score is feeling more prepared.  Same timings as for knowledge scale, but focus of scale varied: TI on resus decision; T2 on choice of birth hospital; T3: on decision to breastfeed.  I:  T1: 76.0;  T2: 76.3;  T3: 68.9;  C:  T1: 52.3;  T2: 54.4;  T3: 54.2;  P values not reported but I higher than C at all 3 time points (at least p<0.05). |  | **Knowledge:**  Favours animation.  **Attitudes & Cognitions:**  Favours animation. | |
| Gagne, 2019, Canada (85) | RCT | Adults with atrial fibrillation, hospital department in Quebec.  No statistically significant difference in education level | I: mean age 57 (SD 13); C: 56 (13)  Males: I: 77%; C: 60%. | N= 60  I= 30  Video animation + face to face education session from nurse (45 minutes)  C= 30  Face to face education session from nurse (45 minutes) | Video on AF, including normal heartbeat, symptoms of AF, risk factors for AF, complications of AF, and treatment options (ie same topics as in the f2f session).  Length: 8 minutes.  Suggests video watched once only.  Link NR. | I n=30; C n=30 for all outcomes.  Knowledge: knowledge of AF test (KAF), focussed on symptoms, disease, impact, risk of stroke. 25 items. Range 0-25 (higher is better). Assessed at T1 (pre-education baseline), T2 (immediately after education); T3 (1 month later); T4 (3 months later).  T2-T1 change:  I: +3.8 (95% CI 2.5-5.0);  C: +1.5 (0.2-2.8);  p=0.014.  T3-T2 change:  I: +0.1 (-0.8-1.1);  C: +1.6 (0.7-2.5);  p=NS.  T4-T3 change:  I: 0.0 (-0.8-0.8);  C: 0.5 (-0.3-1.3).  p=NS. | | Quality of Life (the AFEQT), (Overall score 0-100):  T3 —T1 change:  I: 12.4 (5.0-19.9);  C: 13.6 (6.1-21.0);  p=NS.  T4 – T3 change:  I: 8.7 (0.9-16.6);  C: 3.0 (-4.9-10.9);  p=NS. | Not assessed. | **Knowledge:** Favours animation at T2, no difference at T3 and T4.  **Attitudes & Cognitions:** No difference between arms. | |
| Glanz 2024, USA (49) | RCT | Patients receiving long-term  opioid therapy (LTOT), recruited from dispensing pharmacies.  No statistically significant difference in education level | Age: 60.2 (12.5)  Males: 36.2% | N=1,004  I= 519  animated educational video + usual care  C= 485  Usual care | Participants received a link to a web-based,  6 min animated educational video. Patients had to play the video in its entirety at T0 (baseline).  One month later, participants received an email with a three additional video links to share with family, friends, or caregivers. | Prescription Opioid Overdose Knowledge  Scale (Rx-OOKS) 25 item scale (Higher Rx-OOKS  score represents greater knowledge):  Baseline:  I: 19.3 (95% CI 18.9 to 19.8) (n=515);  C: 11.4 (11.0 to 11.8) (n=481);  4 months  I: 14.8 (14.4 to 15.1) (SD = 3.9) (n=472);  C: 13.2 (12.8 to 13.6) (SD = 4.3) (n=451);  t = 5.926 (p<.0001).    8 months:  I: 15.5 (15.1 to 15.8) (SD = 3.9) (n=468);  C: 14.0 (13.6 to 14.3) (SD = 3.7) (n=438);  t= 5.930 (p<.0001).  Study arm by time interaction: p<0.001. | Not assessed. | | Opioid  risk behaviour  Baseline:  I: 59.9 (55.7 to 64.1) (n=515);  C: 52.0 (47.5 to 56.4) (n=481).  4 months  I: 44.4 (40.0 to 48.8) (SD = 48.7) (n=472);  C: 39.0 (34.6 to 43.5) (SD = 48.2) (n=451);  t = 1.692 (p=NS).  8 months:  I: 43.4 (38.9 to 47.8) (SD = 49.1) (n=468);  C: 38.8 (34.3 to 43.3) (SD = 48.0) (n=438);  t = 1.425 (p=NS).  Naloxone intake at 12 months:  I= 10%;  C= 8.9%  p=NS. | **Knowledge:** Favours animation.  **Behaviour:**  No difference between arms. |  |
| Indradat, 2013, Thailand (58) | RCT | Children with allergic rhinitis.  Education level NR | Age: I: 8.6 (2.6); C: 8.7 (3.1)  Males: I: 77.5%;  C: 50% | N= 80  I= 40  Animated video  C= 40  Oral teaching by 2 HCPs.  Length; 6 minutes | 6 minutes animated video of definition, clinical features & treatment of AR; allergen avoidance; method of using INCS).  Children had to demonstrate the 5 steps of using INCS. If they were unable to do it correctly, the video or the oral instructions were repeated followed by a test, and repeat.  Link NR. | Not assessed. | | Not assessed. | I n=30; C n=30 for all outcomes.  Competence in using inhaler:  Competence after 1^st^ viewing / instruction: I: 57.5%; C: 27.5%; OR = 3.6 (95% CI 1.4-9.1); p=0.007.  Competence after second viewing/education (cumulative %):  I: 95%; C: 60%; OR =12.7 (95%CI 2.7 – 60.1); p=0.004. | **Behaviour:** Favours animation. | |
| Jones 2016, New Zealand (86) | RCT | Patients with acute coronary syndrome.  Education level NR | Age: 60.9 (10.2)  Male:70% | N= 70  I= 35 Animated video + Usual care  C= 35  Usual care | 15 minutes, 2 parts animated video watched on iPad: (1) pathogenesis of acute coronary syndrome and resultant effects; (2) informing patients about behaviours to maintain health.  Patients were given a website link where they could view the intervention again if desired.  Link NR. | Knowledge:  Naming adherence as heart healthy behaviour (post-intervention):  I: 55% (18/33) (n=33);  C: 29% (9/31) (n=31);  p=0.039.  No difference between groups on naming 3 other heart healthy behaviours.  Naming adherence as heart healthy behaviour (7 weeks follow-up):  I: 50%(15/30) (n=30);  C: 27%(8/30) (n=30);  p=NS.  No difference between groups on naming 3 other heart healthy behaviours. | | Illness Perceptions:  (post-intervention):  I: beliefs +0.4 (95% CI -0.2 to 1.0) (n=33);  C: beliefs -0.6 (95% CI -1.2 to 0.0) (n=33); p=0.025.  No differences between groups on the other 8 items of the IPQ.  7 weeks follow-up:  Intervention group had greater improved changes in scores on 3/9 items (p<0.05) compared to Control group.  No difference in change scores between Intervention and Control groups on other 6/9 items.  Medication beliefs:  (Post-intervention):  Intervention greater change in specific concerns:  I: -1.45 (95% CI -2.5 to -0.4) (n=33);  C: -0.3 (95% CI -0.8 to 1.3) (n=33);  p=0.031.  Intervention greater change in general-harm beliefs:  I: -0.5 (95% CI -1.2 to 0.2);  C 0.6 (95% CI -0.1 to 1.3);  p=0.032.  7 weeks follow-up, no differences between Intervention and Control groups. | Return to work: no difference in number of days before return to work, p=NS  Return to normal activities  (number of days):  I: 17.4 (10-9 to 23.8) (n=31);  C: 26.4 (20.7 to 32.1) (n=30); p=0.043.  Exercise taken (minutes until 7 weeks follow-up):  I: +46.6 (7.6 to 82.5) (n=31);  C: -9.2 (-48.2 to 29.7) (n=30); p=0.049.  Self-reported medication adherence (7 weeks follow-up):  I: 46.9 (45.3 to 48.5) (n=31);  C: 47.1 (45.4 to 48.7) (n=30);  p=NS.  Cardiac Anxiety avoidance:  7 weeks follow-up:  Intervention group had lower scores:  I: 6.6 (95% CI 5.0 to 8.1) (n=31);  C: 8.9 (95% CI 7.3 to 10.4) (n=30);  p=0.038. | **Knowledge:** Favours animation on 1 measure. No difference between arms on other 7 measures.  **Attitudes & Cognitions:**  Favours animation on 4/18 Illness Perception items. No difference between arms on other Illness Perception items**.**  Favours animation on 2/4 medication beliefs. No difference between arms on other items**.**  Favours animation on cardiac anxiety avoidance.  **Behaviours:** Favours animation on 2 / 5 measures.  No difference between arms on 3 / 5 measures**.** | |
| Jones 2019, New Zealand (36) | RCT (3 Arms) | Colorectal & gynaecology oncology surgery patients.  Education level NR | Age: 58.7 (16.5)  Male: 37% | N= 96  I1= 33  Animations+ Usual care  I2= 32  Active control (same as intervention but without animations) + Usual care    C2= 31  Usual care | 10 minutes animated video to improve Postoperative Mobilization which described the purpose of early mobilisation, the importance of early oral nutrition, and the  link between these two behaviors (actors filmed plus anatomical 2D and 3D).  Delivered on a computer tablet at their bedside, frequency NR.  Link:  https://youtu.be/1jmgAb1OTew | Not assessed | | I n=33; I2 n=32; C2 n=31 for all outcomes.  Perceptions of surgery and recovery, perceptions of early mobilisation and early oral nutrition and traditional surgery recovery beliefs: (all p=NS).  Quality of recovery:  (I1 vs C) p=NS. | Not assessed | **Attitudes & cognitions:** No difference between the 3 groups in Perceptions of surgery and recovery, perceptions of early mobilization and early oral nutrition and traditional surgery recovery beliefs and Quality of recovery. | |
| Kaewin, 2024, Thailand (41) | Quasi-RCT | Children with epilepsy.  No statistically significant difference in education level | Age: I: 11.0 (0.9); C: 11.0 (0.8)  Males: I: 38%; C: 61.9% | N= 42  I= 21  2 video animations on epilepsy followed by discussion with researcher  C= 21  Usual care (meeting with physician to discuss care) | Two cartoon animations (‘Self-care when I have epilepsy’ 15 minutes, and ‘Anyone can do it’ 7 minutes).  Viewed once only in clinic.  Link NR. | Not assessed. | | Not assessed. | I n=21; C n=21 for all outcomes.  Self-care behaviours (measured on the Self-Care Behaviours Questionnaire for Children with Epilepsy): range 24-96 (higher is better) mean (SD).  8 weeks after intervention:  I: 78.1 (11.4);  C: 65.2 (8.0); p<0.001.  3 sub-scales on medicine taking; avoiding seizure triggers; self-care of abnormal symptoms, all higher in I group (all at least p=0.024). | **Behaviours:** Favours animation**.** | |
| Kayler 2020, USA (57) | RCT | Children and adults with range of kidney problems, Erie County Medical Centre (New York).  No statistically significant difference in education | Age: 60 (NR)  Male:  68% (patients only) | N= 80  I= 42  Animation + audio recorded standard transplant nurse education  C= 38  Audio recorded standard transplant nurse education | 2.16 minutes educational video animation about high kidney donor profile index (KDPI) followed by a 2.05-minute animation about increased risk donor (IRD) in addition to audio recorded standard transplant nurse education.  Watched once.  Link:  <https://www.youtube.com/channel/UC3xXkG9VO83Bkj-jS09Vd3Q/videos> | I n=42; C n=38 for all outcomes.  Patient knowledge mean:  I: 7.4;  C: 6.6;  p=0.036 (β = 0.23; 95% CI, 0.1-1.8). | | Patient Decisional Self-efficacy about KDPI, mean (SD):  I: +1.6 (1.0);  C: +1.2 (1.2);  p=NS.  IRD kidneys mean(SD)  I: +1.46 (0.98);  C: +1.16 (1.35);  p=NS. | Patient IRD Willingness mean (SD): I: 0.7 (1.0); C: 0.05 (1.0);  p=0.003.    Patient KDPI Willingness or Consent (Willingness to accept a KDPI >85%) mean (SD): I:−0.5 (1.3) C: −0.5(1.3); p=NS.  Proportion of subjects signing consent to receive KDPI >85% kidney:  I: 27.5%;  C: 13.5%;  p=NS. | **Knowledge:**  Favours animation.  **Attitudes & cognitions:**  No difference.  **Behaviours:**  Favours animation for IRD willingness only. | |
| Kocaaslan, 2025, Turkey(99) | RCT | Children  with asthma, aged 7-11 years.  Education level NR but all are school children.  No statistically significant difference in parents’ education level | Age:  I: 8.4 (1.4);  C: 8.7 (1.3)  Male:  I:64%;  C:51% | N=93  I= 46  Animated video education + Usual care (verbal instructions and fact sheets)  C= 47  Usual care | The animated education video “Living with Asthma”(LIWA) lasted 7.39 minutes, spoken in Turkish. The “LIWA,” can be viewed on a tablet, accessible via phone, e-mail, or a compact disc (CD).  Link:  https://www.youtube.com/watch?v=OfC83LHBgUM&ab_channel=esrako  caaslan | Not assessed. | The paediatric quality of life inventory (PedsQL) (range 0 to 100) mean(SD):  1 month:  I:58.22 (12.76) (n=42);  C:56.05 (10.02) (n=45);  p=NS.  3 months:  I:69.88 (12.89) (n=39);  C:56.28 (11.24) (n=39);  p=0.007. | | Inhaler technique skill score (median (IQR):  1 month:  I: 6 (4-8) (n=42);  C: 5 (0-8) (n=45);  p<.001.  3 months:  I: 8 (5-10) (n=39);  C: 4 (0-10) (n=39);  p<.001. | **Attitudes & Cognitions:** Favours animation at 3 months but not at 1 month.  **Behaviour:**  Favours animation at 1 month and 3 months. |  |
| Li 2019, China (87) | RCT | Participants with lung cancer, thoracic surgery department of a large tertiary academic medical centre in Southern China.  No statistically significant difference in education level | Age: 53 (9.2)  Male: 59% | N= 80  I= 40  Animation education  C=40  Usual care (face-to-face education) | Standard animation intervention consisted of 3 sections totalling 31 minutes. These sections  Included a 6-minute animation introduction, a 10-minute nurse demonstration, and a 15-minute patient teach-back demonstration. Animation.  Watched twice each day at the patients’ bedside.  Link NR. | Change in score of training related knowledge mean (SD)  I: 73.3 (11.2) (n=40);  C: 63.8 (9.3) (n=40);  The change in the mean score of training-related knowledge (covariates control) OR = 3.22, (95%CI 1.23 to 8.40), p=0.017. | |  | Change in score of exercise compliance (OR, 95%CI):  OR = 1.42, (95%CI 0.56 to 3.64), p=NS. | **Knowledge:**  Favours animation.  **Behaviours:**  No difference between arms. | |
| McIntyre, 2023,Australia (69) | RCT | Patients with Atrial fibrillation (AF) attending outpatient cardiology clinics.  No statistically significant difference in education level | Age: 65.0 (12.2)  Male:65.2% | N= 208  I= 104  Video intervention + Usual care (routine clinical care)  C= 104  Usual care | Four videos on AF. Participants watched all 4 video animations in order immediately after  baseline data collection.  Participants were emailed links to review the video series weekly. Ongoing engagement with the  Intervention was determined by participants and not a requirement of study participation. Videos are  freely available. The length of the videos were between 1.46 to and 5.45 mins.  Link:  https://vimeo.com/showcase/7683573 | Knowledge (16-item questionnaire): OR (95% CI):  2 days post intervention:  I vs C  1.11 (0.92-1.34) (I n=95; C n=92);  p= NS.  90 days post intervention:  I vs C  1.23 (1.01-1.49) (I n=93; C n=92);  p= 0.04. | Satisfaction: OR (95% CI):  2 days post intervention:  Satisfaction with clinical care:  I vs C  1.15 (0.62-2.16); (I n=95; C n=92);  p=NS.  Satisfaction with AF education:  I vs C  1.32 (0.71-2.44); (I n=95; C n=92);  p=NS.  Motivation to maintain medication  adherence: OR (95% CI):  90 days post intervention  I vs C  1.04 (0.88-1.23); (I n=93; C n=92);  p=NS. | | Not assessed. | **Knowledge:**  Favours animation at 90 days, no difference at 2days.  **Attitudes & Cognitions:**  No difference between arms. |  |
| Nana, 2024, Cameroon (40) | RCT | Adults with hypertension (aged <85).  Education level NR | Age: I: 56.4 (10.5); C: 56.6 (11.0).  Males: I: 50.9%; C: 63.2% | N= 110  I= 57  Video animation + Usual care (paper-based and oral clinical counselling)  C= 43 Usual care | Cartoon animation.  Length approximately 10 minutes.  Accessed as often as needed.  Paper-based information takes approximately 10 minutes to read.  Link NR. | Not assessed. | | Not assessed. | Adherence (Morisky scale):  I: Good 20.5%; medium 30.8%; low 48.7%.  (n=39).  C: Good 11.6%; Medium 46.5%; Low 41.9%. (n=43).  p=NS. | **Behaviour:** No difference between arms. | |
| Saengow, 2018, Thailand (56) | RCT | Paediatric patients who were diagnosed with epilepsy who have been visiting the routine service paediatric neurology clinic at Maharat Nakhon Ratchasima Hospital, Thailand.  Education level NR | Age: I: 7.6 (4.5); C: 7.6 (4.8)  Male: 58% | N= 214  I= 126  Video + advice  C= 88  Advice only | 3 minutes animated video about appropriate use of anti-epileptics.  Parents watched the video once via a portable DVD player.  Link:  <https://www.youtube.com/watch?v=uEHmvl9qZlc> | Knowledge, mean change in score pre-post immediately post-intervention:  I: +0.5 (n=126);  C: +0.1 (n=88);  p<0.001.  Knowledge, mean change in score pre-post 3 months post-intervention:  I: +0.6 (n=126);  C: +0 (n=88);  p<0.001. | | Not assessed | Change in drug adherence:  I: 42.9% (n=126);  C: 15.9% (n=88);  p<0.001. | **Knowledge:** Favours animation.  **Behaviour:** Favours animation. | |
| Schroeder, 2021, USA (55) | RCT | Adults with urinary incontinence, recruited at specialist hospital department.  Education level NR | Age: I: 59.7 (13.7);  C: 60.5 (16.4)  Male: NR | N= 98  I= 50  Video (animation followed by video vignettes). Could ask questions afterwards if needed  C= 48 Traditional face-to-face counselling from doctor. Could ask questions afterwards if needed | Animated videos created with separate English and Spanish versions.  Comprising 2 components: animated diagrams and models as an  educational foundation with voiceover explanation, followed by short vignettes depicting a common exchange between a patient  and a physician when seeking care for stress and urgency urinary  incontinence.  Length: 7.5 minutes.  Viewed only once.  Link:  http://links.lww.com/  FPMRS/A178 | I n=50; C n=48 for all outcomes.  Knowledge mean (SD):  T1 (Pre-information): I: 45.0 (27.7); C: 40.1 (25.6); p=NS.  T2 (immediately after education): I: 73.0 (25.7); C: 63.0 (30.9); p=NS.  T3 (6-8 weeks later): I: 68.3 (29.3); C: 63.3 (29.8); p=NS. | | Quality of Life (Incontinence Impact questionnaire (IIQ) -7)mean(SD).  T2: I: 8.3 (5.8); C: 9.0 (5.4);  p=NS.  T3: I: 9.1 (5.7); C: 7.6 (5.9);  p=NS.  Satisfaction (with care), 10cm VAS.  T2: I; 9.7 (1.7); C: 9.5 (1.4);  p=NS. | Not assessed. | **Knowledge:** No difference between arms.  **Attitudes & Cognitions:** No difference between arms. | |
| Sommer, 2022, Switzerland (89) | RCT | Adults with keratoconus, recruited at hospital corneal clinic.  Education level NR | Age: I: 31.1 (12.2); C: 26.9 (10.8)  Males: I: 82%; C: 81% | N= 43  I= 22  Video animation + Usual care (face to face consultation)  C= 21  Usual care | Animated scenes giving information on keratoconus.  Length 5 minutes.  Viewed once only.  Link:  www.youtube.com/  watch?v=9oWeP137x (but no longer available) | Knowledge (details of measure not reported): assessed immediately after the consultation.  12.0% (95% CI 5.8%-18.2%; p<0.001). (I n=22; C n=21). Compared to the control, knowledge was 12% higher in the intervention group. | | Not assessed. | Not assessed. | **Knowledge:** Favours animation. | |
| Wonggom 2020, Australia (66) | RCT | Patients with heart failure patients, 3 outpatient clinics in three public hospitals in South Australia.  No statistically significant difference in education level | Age: 67.5 (11.3)  Male: 81% | N= 36  I= 17  Avatar app + usual care  C= 19  Usual care | Avatar app which is based on the Heart Foundation of Australia's booklet, ‘Living Well with Heart Failure’.  Watched on tablet computer. Patients could watch as often, or as little as they would like during the clinic appointment.  Link NR. | I n=17; C n=19 for all outcomes.  Knowledge of heart failure (%), change in score pre-post at 30 days post-Int:  I: +15.3%;  C: +3.5%;  p=NS.  Knowledge of heart failure (%), change in score pre-post at 90 days post-Int:  I: +22.2%;  C: +3.7%;  p=0.002. | | Self-care confidence, change in score pre-post at 30 days post-Int:  I: +7.4%;  C: +4.9%;  p=NS.  Self-care confidence, change in score pre-post at 90 days post-Int:  I: +19.6%;  C: +5.0%;  p=NS. | Self-care maintenance, change in score pre-post at 30 days post-Int:  I: +6.4%;  C: +7.0%;  p=NS.    Self-care behaviours, change in score pre-post at 90 days post-Int:  I: +11.0%;  C: +6.8%;  p=NS. | **Knowledge:** Favours animation at 90 days; no difference between arms at 30 days.  **Attitudes & Cognitions:** no difference between arms.  **Behaviours:** No difference between arms. | |
| Ye, 2023, China (24) | RCT | Adults following gastrointestinal surgery (usually for colorectal cancer).  Education level NR | Age: I: 57.0 ( 7.5); C: 57.6 (7.4)  Males: I: 62%; C: 58% | N= 66  I= 33  Animation + individual guided pelvic floor muscle guidance  C= 33  Individual guided pelvic floor muscle guidance. | 3D animation of pelvic floor muscle and bone.  Viewed only once in clinic.  Length not reported.  Link NR. | Not assessed. | | Not assessed. | I n=32; C n=31 for all outcomes.  Training completion (rated as optimal/good/poor):  T1 (pre-op, post intervention):  C: 3/16/12; I: 12/14/6;  p=0.023.  T2 (1 week post-surgery):  C: 4/15/12; I: 13/13/6;  p= 0.023.  T3 (2 weeks post-surgery): C: 6/15/10; I: 15/13/4;  p=0.006.  T4 (1 month post-surgery):  C: 5/15/11; I: 14/14/4.  p=0.023.  T5 (3 months post-surgery): C: 4/12/15; I: 11/13/8;  p=NS. | **Behaviours:** Favours animation at 4 of the 5 follow-up periods. (1 out of 5 no difference between arms). | |

I= Intervention, C= Control, NR=Not reported, NS= Not significant, RCT=Randomised Controlled Trial
